# Benchmarking fMRI Denoising Pipelines

**DOI:** 10.1002/hbm.70561

**Published:** 2026-07-01

**Authors:** Tianye Zhai, Hong Gu, Anika Holton, Elanor Chang, Blaise B. Frederick, Thomas J. Ross, Yihong Yang, Amy C. Janes

**Affiliations:** ^1^ Neuroimaging Research Branch, Intramural Research Program National Institute on Drug Abuse, National Institutes of Health Baltimore Maryland USA; ^2^ Department of Psychiatry Harvard University Medical School Boston Massachusetts USA

**Keywords:** 1‐step regression, fMRI denoising pipeline, multi‐echo, pre‐whitening, time‐shifted physiological confounds

## Abstract

Functional magnetic resonance imaging (fMRI) is a powerful tool for probing neuronal activity in vivo, but fMRI data are inherently noisy. To mitigate this, a wide range of denoising strategies have been developed, including volume censoring, anatomical component‐based noise correction (aCompCor), ICA‐based methods (e.g., AROMA, FIX), and multi‐echo approaches (e.g., ME‐ICA, tedana). These techniques are often applied in different combinations and have been predominantly evaluated on single‐echo resting‐state fMRI data—typically without incorporating more recent methodological advances known to improve modeling, such as order‐independent “1‐step regression”, modeling temporal autocorrelation (pre‐whitening), and temporal shifting of physiological nuisance regressors. To fill this gap, we used a framework that incorporates these methods and benchmarked a range of denoising pipelines across task and resting‐state, single‐ and multi‐band, and single‐ and multi‐echo fMRI datasets, using different combinations of standard denoising confounds. Pipeline performance was evaluated using temporal signal‐to‐noise ratio (tSNR) and percentage remaining degrees‐of‐freedom (DoF), effectiveness of motion correction, and effectiveness of signal preservation. While pipelines only using ICA were insufficient, those that incorporated physiological nuisance regressors performed well. Additional improvements were observed when temporally shifted physiological regressors were accounted for. Based on these results, we provide recommendations for selecting denoising pipelines and emphasize the need for continued benchmarking as new methods are developed or applied in novel contexts.

## Graphical abstract

This paper benchmarked fMRI denoising pipelines on task‐ and resting‐state, single‐ and multi‐echo fMRI datasets, in a modern 1‐step regression framework, with pre‐whitening and time‐shifted physiological confounds involved.

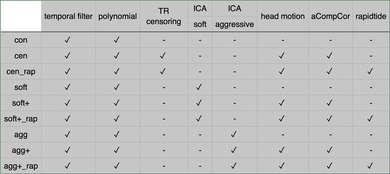

## 1 Introduction

Despite being an indispensable tool for studying human brain function, functional magnetic resonance imaging (fMRI) data are inherently noisy, with the BOLD signal reflecting neuronal activity accounting for only a small fraction of the total signal fluctuations (Bianciardi et al. 2009; Caballero‐Gaudes and Reynolds 2017). Identifying and suppressing noise is therefore critical to enhance the detection of neuronal signals in fMRI data. However, there is currently no consensus on the optimal denoising pipeline. A systematic, evidence‐based evaluation is needed to determine the most effective combinations of denoising methods while accounting for methodological considerations, data types (i.e., resting‐state and task‐based fMRI), and various sources of noise. fMRI data are affected by multiple noise sources, including head motion, physiological confounds, and methodological artifacts unrelated to neurobiological signals but instead arising from how the data are acquired, processed, and modeled. A wide range of denoising strategies have been developed to address these challenges. In what follows, we briefly outline these strategies before addressing the key question of how different methodological combinations impact data quality, as assessed using metrics such as the temporal signal‐to‐noise ratio (tSNR) and percentage remaining degrees of freedom (DoF), motion correction effectiveness, and empirical measures of functional connectivity and task activation.

Among the various sources of noise in fMRI data, head motion is one of the most detrimental (Caballero‐Gaudes and Reynolds 2017). Motion during the scanning sequence can cause not only spatial misalignment between volumes, but also artifacts during acquisition that contaminate the signal (e.g., spin‐history effects) (Friston et al. 1996). Head motion is typically estimated by registering each fMRI volume to a reference volume, yielding 6 directional rigid body transform parameters (3 translational and 3 rotational). These parameters, along with their temporal derivatives and, in some cases, quadratic terms, are used as regressors in denoising pipelines (Johnstone et al. 2006; Satterthwaite et al. 2013; Van Dijk et al. 2012). Further, Power et al. (2012) demonstrated that the frame‐to‐frame displacement (i.e., the relative movement between consecutive volumes) negatively impacts both time series and the functional connectivity estimates in resting‐state fMRI, even after motion parameter regression. As a result, censoring (or “scrubbing”) time points with excessive frame‐to‐frame displacement, has become a widely adopted strategy, with over 7000 citations. Another widely used approach is independent component analysis (ICA)‐based denoising, such as ICA‐FIX (Salimi‐Khorshidi et al. 2014) and ICA‐AROMA (Pruim, Mennes, van Rooij, et al. 2015). These techniques use spatial ICA to decompose whole‐brain fMRI into independent components that are classified either manually or automatically to identify and remove noise‐related components.

In addition to motion, physiological processes such as cardiac pulsation and respiration pose as another major source of noise that can contaminate the fMRI signal (Caballero‐Gaudes and Reynolds 2017). Early denoising approaches regressed out averaged time courses from tissues less likely to have neuronal activity, such as cerebrospinal fluid (CSF) and white matter (WM) (Fox and Raichle 2007; Fox et al. 2005; Parkes et al. 2018; Rombouts et al. 2003). More advanced methods, such as the anatomical component‐based noise correction (aCompCor), estimate and regress out high‐variance ranking principal components derived from WM and/or CSF time courses, improving performance relative to the WM/CSF mean regression (Behzadi et al. 2007; Muschelli et al. 2014). Importantly, some physiological noise sources (e.g., end‐tidal CO_2_ and cerebral blood flow) exhibit temporal delays relative to the fMRI signal, underscoring the need to incorporate temporal lags when modeling these noise components (Bright and Murphy 2013; Bright et al. 2017). To address this, phase‐shifted WM, CSF, and soft tissue time series were employed to improve the modeling of the global physiological noise (Anderson et al. 2011). Techniques such as regressor interpolation at progressive time delays (RIPTiDe; rapidtide) have been developed to estimate the maximum lagged correlation between a probe signal and the whole‐brain fMRI time series to create voxel‐wise‐optimized nuisance regressors (Erdogan et al. 2016; Tong and Frederick 2010).

Multi‐echo acquisition has enabled the development of multi‐echo‐based fMRI denoising. Multi‐echo fMRI acquires MRI signals at multiple echo times (TE) following each excitation pulse (Posse 2012). Signals acquired at earlier TEs provide higher signal intensity, whereas those from later TEs enhance BOLD contrast. Currently, the most widely used multi‐echo denoising pipeline is “tedana” (developed from the ME‐ICA pipeline), which implements two levels of multi‐echo‐based denoising. The first level involves mathematically combining data from different echoes with different weightings, based on voxel‐specific T2* estimates. This produces a single, optimally combined dataset analogous to one acquired at the TE optimal for each voxel. In the second level of multi‐echo denoising, ICA is applied to the optimally combined dataset to identify spatiotemporal components that are then used to fit the original echo specific data on a voxel‐wise basis. The resulting beta weights are fit to theoretical TE‐dependent and TE‐independent models across the TEs to generate metrics used to distinguish the BOLD and non‐BOLD components of the data. The non‐BOLD components are subsequently removed from the fMRI data (Kundu et al. 2017; Olafsson et al. 2015).

After estimating the nuisance time series, a general linear model (GLM)‐based regression is typically applied to remove these noise components and generate a cleaned dataset (Satterthwaite et al. 2013). Notably, there are many different combinations of these nuisance regressors and many studies have evaluated denoising pipelines (Burgess et al. 2016; Ciric et al. 2017; Dipasquale et al. 2017; Mascali et al. 2021; Parkes et al. 2018; Pruim, Mennes, Buitelaar, and Beckmann 2015; Wang et al. 2024). These studies provide valuable insights into pipeline selection. However, benchmarking that incorporates several important methodological advances including 1‐step regression, pre‐whitening, and temporally shifted physiological regressors is lacking.

In the current study, we evaluated fMRI denoising pipelines using a framework that incorporates these modern technological considerations. We evaluated pipelines that include a wide range of nuisance regressors, including motion parameters, aCompCor, ICA‐AROMA, volume censoring, and more recent methods such as ME‐ICA/tedana for multi‐echo‐based denoising, and rapidtide to model temporally shifted physiological confounds. To provide a modern and methodologically robust evaluation of fMRI denoising pipelines, our approach adopts a benchmarking framework that incorporates several current best practices in GLM‐based nuisance regression (Bright et al. 2017; Olszowy et al. 2019). Specifically, all pipelines were implemented using a 1‐step regression approach, in which all nuisance regressors are entered simultaneously into a single GLM (Lindquist et al. 2019). This design avoids re‐introducing noise in sequential denoising GLMs and concerns related to the order of sequential regressions, improves the stability of model estimates, and allows for proper accounting of degrees of freedom (Lindquist et al. 2019). In addition, we applied pre‐whitening in our GLM benchmarking to model temporal autocorrelation (Olszowy et al. 2019; Woolrich et al. 2001) and included temporally shifted nuisance regressors where appropriate to model physiological noise (Bright and Murphy 2013; Bright et al. 2017). Together, these methodological choices address gaps in the literature, and improve the interpretability and validity of denoising pipeline comparison.

Our benchmarking framework further extends prior efforts by including both resting‐state and task‐based fMRI data, allowing us to directly assess the impact of denoising on task activation—an area that is commonly overlooked (Burgess et al. 2016; Ciric et al. 2017; Dipasquale et al. 2017; Parkes et al. 2018; Pruim, Mennes, Buitelaar, and Beckmann 2015). For resting‐state data, we employed high‐pass filtering (0.01 Hz‐Nyquist) as opposed to the conventional band‐pass filtering (0.01–0.1 Hz). This choice reduces the loss of DoF associated with band‐pass filtering in modern acquisition protocols with higher temporal resolution (Caballero‐Gaudes and Reynolds 2017; Hallquist et al. 2013) and avoids the tendency of losing the signal of interest at higher frequency bands that might be neuronal related (Boubela et al. 2013; Chen and Glover 2015; Griffanti et al. 2014; Lee et al. 2013; Pruim, Mennes, van Rooij, et al. 2015). Finally, by including single‐ and multi‐band, single‐ and multi‐echo datasets, our framework enables evaluation across a wider range of acquisition protocols. Together, these methodological enhancements allow for a more comprehensive and representative comparison of denoising pipelines and provide practical guidance for optimizing GLM‐based denoising in contemporary fMRI research.

## 2 Materials and Methods

### 2.1 Participants

Four cohorts of healthy control participants were included in the current study: a cohort of 44 participants who completed a block design fMRI task (Cohort A), a cohort of 34 participants who completed an event‐related task fMRI and a single‐band single‐echo resting‐state fMRI (Cohort B), a cohort of 33 participants who completed a multiband multi‐echo resting‐state scan (Cohort C), and a larger cohort of 847 participants from the Adolescent Brain Cognitive Development (ABCD) study (Casey et al. 2018) with multiband single‐echo acquisition (Cohort D). Cohorts A, B, and C were part of larger studies conducted at the National Institute on Drug Abuse Intramural Research Program (NIDA‐IRP) in Baltimore, Maryland. Those studies were approved by the NIDA‐IRP Institutional Review Board, and written informed consent was obtained from each participant. Participants were excluded if they had any major medical, neurological, or psychiatric illnesses.

### 2.2 MRI Acquisition

For Cohorts A–C, MRI scans were conducted using Siemens 3 T scanners (Trio/Prisma; Siemens, Erlangen, Germany). For Cohort A, three runs of whole‐brain blood‐oxygen‐level‐dependent (BOLD) fMRI data with 212 volumes were acquired using a single‐shot, echo‐planar imaging (EPI) sequence (TE = 27 ms, TR = 2 s, flip angle = 78°, spatial resolution = 3.44 mm × 3.44 mm × 4 mm, no gap), while participants performed an n‐back working memory task. For Cohort B, five runs of whole‐brain BOLD fMRI data with 144 volumes were acquired during a flanker task, along with one resting‐state run with 240 volumes with eyes open, using the same EPI sequence as Cohort A. For Cohort C, one run of resting‐state fMRI data with 452 volumes was acquired using a single‐shot, multiband (factor 4), multi‐echo EPI sequence (TE = 12.60/29.16/45.75/62.28 ms, TR = 1.33 s, flip angle = 67°, spatial resolution = 2.51 mm × 2.51 mm × 2.5 mm, no gap), with eyes open. For Cohorts A through C, anatomical images were acquired using a T1‐weighted 3D magnetization‐prepared rapid gradient‐echo sequence (MPRAGE) sequence (TE = 3.51 ms / 3.42 ms, TR =1.9 s, flip angle = 9°, spatial resolution = 1 mm × 1 mm × 1 mm). For Cohort D, details of the scanning parameters are detailed in prior work (Casey et al. 2018). Five modalities of fMRI data were analyzed across the four cohorts in this study: block design task‐based fMRI (bd‐tfMRI), event‐related task‐based fMRI (er‐tfMRI), single‐band single‐echo resting‐state fMRI (sbse‐rsfMRI), multiband multi‐echo resting‐state fMRI (mbme‐rsfMRI), and multiband single‐echo resting‐state fMRI (mbse‐rsfMRI). Details on the task designs can be found in the Supporting Information.

### 2.3 Analytical Approach

#### 2.3.1 Image Preprocessing

For Cohorts A–C, preprocessing was conducted using fMRIPrep (v.23.1.4, https://fmriprep.org/en/23.1.4/), which involved discarding the first two volumes to account for T1 non‐equilibrium, slice‐timing correction, volume registration, head motion estimation, and spatial normalization to MNI space at 2 mm isotropic resolution. For Cohort D, preprocessing was conducted using fMRIPrep (v.25.1.4, https://fmriprep.org/en/25.1.4/), which involved discarding the first adaptive number of volumes to account for the T1 non‐equilibrium based on automatic detection, slice‐timing correction, volume registration, head motion estimation, and spatial normalization to MNI space at the native resolution of 2.4 mm isotropic in the ABCD dataset.

#### 2.3.2 Noise Regressor Estimation

Several sets of regressors were estimated from the preprocessed fMRI data using different noise estimation programs and packages as follows:

1. The 6 directional head motion parameters (3 translational and 3 rotational) were estimated with rigid‐body volume registration (*mcflirt*, FSL) contained within the fMRIPrep pipeline. The three rotational dimensions were then transformed into degrees from radians (*1deval*, AFNI), which served as the motion regressors for further use. Head motion was also evaluated at the frame‐to‐frame level to further control image quality by using relative displacement calculated between consecutive volumes using the Euclidean norm (Enorm) (*1d_tool.py*, AFNI). Volumes with Enorm > 0.35 mm were labeled for censoring along with the volumes from the previous TR.
2. ICA based motion noise regressors were estimated using FSL (v.6.0.5, https://www.fmrib.ox.ac.uk/fsl) and ICA‐AROMA (v.0.4.4, https://github.com/maartenmennes/ICA‐AROMA). Time courses of noise components were then orthogonalized to time courses of signal components using Matlab (v.2022a, The MathWorks Inc., Natick, Massachusetts). Both the original and orthogonalized time courses of noise components were retained for further use as motion related regressors (AROMA regressors).
3. For multi‐echo data, the EPI data from multiple echoes were first optimally combined using the “t2smap” workflow of tedana (v.23.0.1, https://tedana.readthedocs.io/en/23.0.1/index.html) wrapped within fMRIPrep to generate a mathematically consolidated 4‐D dataset that takes advantage of both the greater signal from the earlier echoes and the better contrast from the later echoes. This produced an optimally combined dataset that was then used as input for all of the compared pipelines. For the multi‐echo ICA related pipelines, the “tedana” workflow of the tedana software package (v.23.0.1, https://tedana.readthedocs.io/en/23.0.1/index.html) was conducted post‐fMRIPrep, combining multi‐echo ICA with an automatic classification procedure to label noise and signal components based on whether they fit with the theoretical T_2_* signal decay to be considered TE dependent, thus teasing apart BOLD and non‐BOLD components of the EPI data. Both the original and the orthogonalized time courses of noise components were retained for further use as multi‐echo based non‐BOLD noise regressors.
4. For physiological noise regressors, the aCompCor (Behzadi et al. 2007) components were estimated as the top four principal components of the signal from the WM and CSF (*3dmaskSVD*, AFNI). Further, a time‐ varying physiological signal that is based on the whole‐brain blood arrival time lag (Tong and Frederick 2010), was estimated with the rapidtide package (v.2.8.2, https://github.com/bbfrederick/rapidtide). The 4‐D voxel‐specific regressor representing the temporal shifted global probing signal according to the voxel‐specific blood arrival time lag was retained for further use. For Cohort D (ABCD dataset), this rapidtide 4‐D regressor underwent a further step of PCA, similar to the procedure of the aComoCor described above. The top four principal components of this rapidtide 4‐D time series within the rapidtide mask were estimated (*3dmaskSVD*, AFNI) to reduce the computational and data storage burden for this large dataset.
5. For the task‐based fMRI data, low‐frequency components (< 0.01 Hz) were modeled in the GLM to control for slow drifts; for the resting‐state fMRI, a set of high‐pass temporal filtering was applied within the frequency range of 0.01 Hz‐Nyquist. Both were implemented with regressors generated via a discrete sine/cosine transform to select frequencies (*1dBport*, AFNI).

#### 2.3.3 Denoising Pipelines and GLM

After all noise regressors were estimated/generated, they were grouped to form different combinations as input to the GLM, each representing different denoising pipelines (9 for single‐echo data and 15 for multi‐echo data‐based pipelines, with an additional 6 multi‐echo ICA‐based pipelines) being compared, as summarized in Table 1 and detailed below:

1. “cen” (Censoring): This represents the motion TR censoring pipeline, which also included the 12‐degree head motion parameters (6 original parameters along with their temporal derivatives) and the aCompCor regressors;
2. “cen_rap” (Censoring and rapidtide): A rapidtide version of the motion TR censoring pipeline, which included an extra voxel‐specific rapidtide regressor (an extra set of rapidtide component regressors for the ABCD dataset) on top of “cen”;
3. “soft” (ICA soft): This represents the ICA‐based denoising pipelines, i.e., AROMA and/or tedana. In these pipelines, the time courses of components identified as noise by AROMA and tedana were orthogonalized to those identified as signal (soft regressors) to perform “non‐aggressive” denoising that prioritized signal preservation;
4. “soft+” (ICA soft plus head motion and aCompCor): This includes the ICA soft regressors along with the 12‐degree head motion parameter and the aCompCor regressors;
5. “soft+_rap” (ICA soft plus and rapidtide): A rapidtide version of the soft+ pipeline, which includes an extra voxel‐specific rapidtide regressor (an extra set of rapidtide component regressors for the ABCD dataset) on top of “soft+”;
6. “agg” (ICA aggressive): Similar to the “soft” pipeline, this represents the same ICA‐based denoising pipelines but uses the aggressive regressors (directly using the noise time courses without orthogonalizing to those of the signal components) to prioritize noise suppression;
7. “agg+” (ICA aggressive plus head motion and aCompCor): This includes the aggressive regressors, as well as the 12‐degree head motion parameter and the aCompCor regressors;
8. “agg+_rap” (ICA aggressive plus and rapidtide): This is a rapidtide version of the agg+ pipeline, which includes an extra voxel‐specific rapidtide regressor (an extra set of rapidtide component regressors for the ABCD dataset) on top of “agg+”;
9. “con” (Control): This serves as a control pipeline, which includes none of the above‐mentioned noise regressors and only the temporal filter and polynomial detrending regressors that are common across all compared pipelines (see details below).

All pipelines also included a set of temporal filter regressors (*1dBport*, AFNI) and a set of polynomial detrending terms determined automatically (3*dDeconvolve*, AFNI).

**TABLE 1:** Regressors included in denoising pipelines.

	Temporal filter	Polynomial	TR censoring	ICA soft	ICA aggressive	Head motion	aCompCor	Rapidtide
con	Yes	Yes	—	—	—	—	—	—
cen	Yes	Yes	Yes	—	—	Yes	Yes	—
cen_rap	Yes	Yes	Yes	—	—	Yes	Yes	Yes
soft	Yes	Yes	—	Yes	—	—	—	—
soft+	Yes	Yes	—	Yes	—	Yes	Yes	—
soft+_rap	Yes	Yes	—	Yes	—	Yes	Yes	Yes
agg	Yes	Yes	—	—	Yes	—	—	—
agg+	Yes	Yes	—	—	Yes	Yes	Yes	—
agg+_rap	Yes	Yes	—	—	Yes	Yes	Yes	Yes

For each denoising pipeline, the estimated regressor sets were used to generate the regression matrix (*3dDeconvolve*, AFNI). A generalized least squares GLM was then conducted using this matrix and the 4‐D dataset as input (*3dREMLfit*, AFNI), with pre‐whitening applied during the nuisance and/or task regression to account for temporal autocorrelation (Bright et al. 2017; Olszowy et al. 2019). All noise regressors, including the rapidtide regressors and polynomial detrending terms, were incorporated into a single GLM step to prevent the re‐introduction of artifacts into the fMRI data (Lindquist et al. 2019). The resulting output was then spatially smoothed using a 6 mm FWHM Gaussian kernel for subsequent analyses.

#### 2.3.4 Evaluation of Different Denoising Pipelines

Three main indices were used to evaluate the denoising pipelines: tSNR and percentage of remaining DoF, effectiveness of motion correction, and signal preservation.

##### 2.3.4.1 Temporal Signal‐To‐Noise Ratio (tSNR) and Percentage of Remaining DoF

The tSNR was defined as: tSNR=meansigpreGLMstdsigpostGLM_Residual, and is commonly used to assess data quality after denoising procedures (Beckers et al. 2023; Dipasquale et al. 2017). As DoF is a crucial factor in regression‐based fMRI denoising, which affects the accuracy of linear estimation (Caballero‐Gaudes and Reynolds 2017; Ciric et al. 2017; Parkes et al. 2018; Pruim, Mennes, Buitelaar, and Beckmann 2015; Pruim, Mennes, van Rooij, et al. 2015), we calculated the percentage of the remaining DoF after the GLM. We then calculated the averaged tSNR within gray matter (GM) across subjects and accompanying the averaged tSNR with the calculated percentage remaining DoF for all the comparing pipelines.

##### 2.3.4.2 Effectiveness of Motion Correction

To evaluate the effectiveness of motion correction, we examined the relationship between head motion and (1) task activation beta weights for the two task‐based fMRI datasets and (2) functional connectome for the three resting‐state datasets after applying the denoising pipelines (Ciric et al. 2017; Dipasquale et al. 2017; Parkes et al. 2018). An effective denoising pipeline for motion correction should theoretically result in task activation amplitude and functional connectome strength that are less influenced by head motion. We defined head motion as the mean Enorm of the frame‐wise displacement for each subject. For the block design n‐back task, we used the beta weights from the 2‐back versus 0‐back contrast. For the event‐related flanker task, we used the incorrect versus correct response contrast from the 1st level analysis for each subject. The decision to use an error‐related contrast, rather than a congruency‐based contrast, was made to challenge the pipelines, as error trials are fewer in number compared to congruent trials.

For the three resting‐state fMRI datasets (single‐band single‐echo, multiband multi‐echo, and multiband single‐echo), we used functional connectome as our measure of interest. We first generated a 411‐ROI volumetric cortical and subcortical atlas in the MNI space (Figures S1 and mmpPlus_411_mni.zip), which is built upon the volumetric version (https://afni.nimh.nih.gov/pub/dist/atlases/MNI_HCP/) of the MMP 360 cortical parcellation (Glasser et al. 2016). This included additional subcortical structures, such as 6 striatal sub‐regions (Tziortzi et al. 2014) from the FSL package, 10 hippocampal and amygdala sub‐regions (Amunts et al. 2005), and 14 thalamic ROIs (Behrens et al. 2003) generated with the Anatomy toolbox (Eickhoff et al. 2005). We also included 17 cerebellar ROIs (Buckner et al. 2011) and 4 ROIs in the globus pallidus (Pauli et al. 2018). The averaged time course of each ROI was extracted, and a 411 × 411 connectome matrix was calculated for each subject per denoising pipeline.

The upper triangle of the matrix was retained, vectorized, and Fisher‐*Z* transformed for each of the subjects. We then calculated the Pearson correlations between head motion and either task activation or pairwise connectome across subjects. These correlations were Fisher‐*Z* transformed, and their absolute values were used to evaluate the effectiveness of motion correction of the pipelines. For Cohort D (ABCD dataset), functional connectome matrices were harmonized across scanning sites prior to head motion correlation, using the CovBat method (Chen et al. 2022; Fortin et al. 2018).

##### 2.3.4.3 Effectiveness of Signal Preservation

Next, we empirically evaluated the signal preservation capabilities of the denoising pipelines. We first evaluated the spatial patterns of task activation and functional connectivity. For the block design task (n‐back task), we used the 2‐ versus 0‐back contrast. The 2‐back versus 0‐back contrast from the 1st level analysis for each subject was then used in the 2nd level analysis to evaluate group activation patterns, using a voxel‐wise one‐sample *t*‐test (*3dttest++*, AFNI). Statistical significance was defined at the voxel‐level *p* < 0.001, with randomization‐based multiple comparison correction at cluster‐level *α* < 0.05, NN1 (face‐wise nearest neighbor). For the event‐related task fMRI dataset (flanker task), we used the error‐related activation contrast (incorrect vs. correct responses). The statistical significance for the 2nd level analysis followed the same criteria as the n‐back task.

For the three resting‐state fMRI dataset (sbse‐rsfMRI, mbme‐rsfMRI, and mbse‐rsfMRI), we conducted the 1st level analysis using the posterior cingulate cortex (PCC) as the seed ROI, defined as a 6 mm radius sphere centered at MNI coordinates [0, −54, 26] (Andrews‐Hanna et al. 2007; Dipasquale et al. 2017; Van Dijk et al. 2010). This ROI was used to calculate the functional connectivity (*3dDeconvolve*, AFNI) resembling the canonical default mode network (DMN) (Greicius et al. 2003; Raichle and Snyder 2007). The resulting connectivity maps for each subject were then Fisher‐Z transformed and used in the 2nd level analysis for the group connectivity pattern. For Cohort D, the Fisher‐Z transformed connectivity maps were harmonized prior to the 2nd level analyses, using the CovBat method (Chen et al. 2022; Fortin et al. 2018). The 2nd level analyses were conducted using voxel‐wise one‐sample *t*‐tests (*3dttest++*, AFNI). Statistical significance was defined at voxel‐level *p* < 0.001 (sbse‐rsfMRI) and *p* < 1e−8 (mbme‐rsfMRI), with randomization‐based multiple comparison correction at the cluster level (*α* < 0.05, NN1). For the mbse‐rsfMRI dataset from Cohort D, we utilized the effect size Cohen's *d* with *d* > 1.2 as the thresholding method for visualizing the connectivity patterns. The change in thresholding across different cohorts is due to the severe changes in statistical sensitivity due to different factors such as multi‐echo, or large sample size. We adopted pragmatical thresholds that yield spatially specific patterns consistently across the majority of the pipelines within the same cohort. These choices of thresholds were mostly made for visualization and comparability purposes only.

We then evaluated the relative task activation and functional connectivity of key ROIs across the different pipelines. We first averaged the 1st level results (task activation contrasts or functional connectivity maps, as described above) across all subjects and pipelines to generate a “mega‐mean” map for each modality. ROIs were then defined using a two‐step approach. First, several key nodes were selected a priori based on canonical task activation and/or resting‐state network patterns (e.g., the executive control network regions for the n‐back task, or the DMN regions for the PCC resting‐state functional connectivity (rsFC)). These predefined key nodes determined which ROIs were included in the subsequent analysis. Then we use the “mega‐mean” map to determine the spatial locus of each ROI within these predefined key nodes. For each pre‐defined key nodes, the locus of local maximum (peak in mean value) within the corresponding cluster was then selected as the ROI coordinates, and a spherical ROI (6 mm radius) was defined around the coordinates.

We then calculated the relative activation and connectivity values for these ROIs across subjects by normalizing the averaged activation/connectivity values within the ROI with the averaged activation/connectivity values within the whole‐brain GM across subjects: relativeValue=(meanROI−meanGM)stdGM, where *relativeValue* refers to relative activation in task‐based fMRI and relative functional connectivity in resting‐state fMRI. For the n‐back task, an additional analysis was conducted to assess the relationship between the task‐related activation or de‐activation of these key node ROIs and working memory task performance for all denoising pipelines. Reaction time and the discriminability index (d‐prime) were used to evaluate behavioral performance on the task. d‐prime takes both hit rate and false alarm rate into account (Haatveit et al. 2010) in assessing working memory performance. Extreme values (0 or 1) of the hit rate/false alarm rate were corrected using the log‐linear method in the d‐prime calculation (Hautus 1995).

For the three resting‐state datasets, our primary analyses used high‐pass filtering as described above. We conducted the above analyses using conventional band‐pass filtering (0.01–0.1 Hz) as a reference. We also calculated the power spectral density of the high‐pass filtered GLM residuals to illustrate power spectral characteristics across pipelines.

## 3 Results

### 3.1 Temporal Signal‐To‐Noise Ratio (tSNR) and Percentage Remaining DoF

**FIGURE 1:**
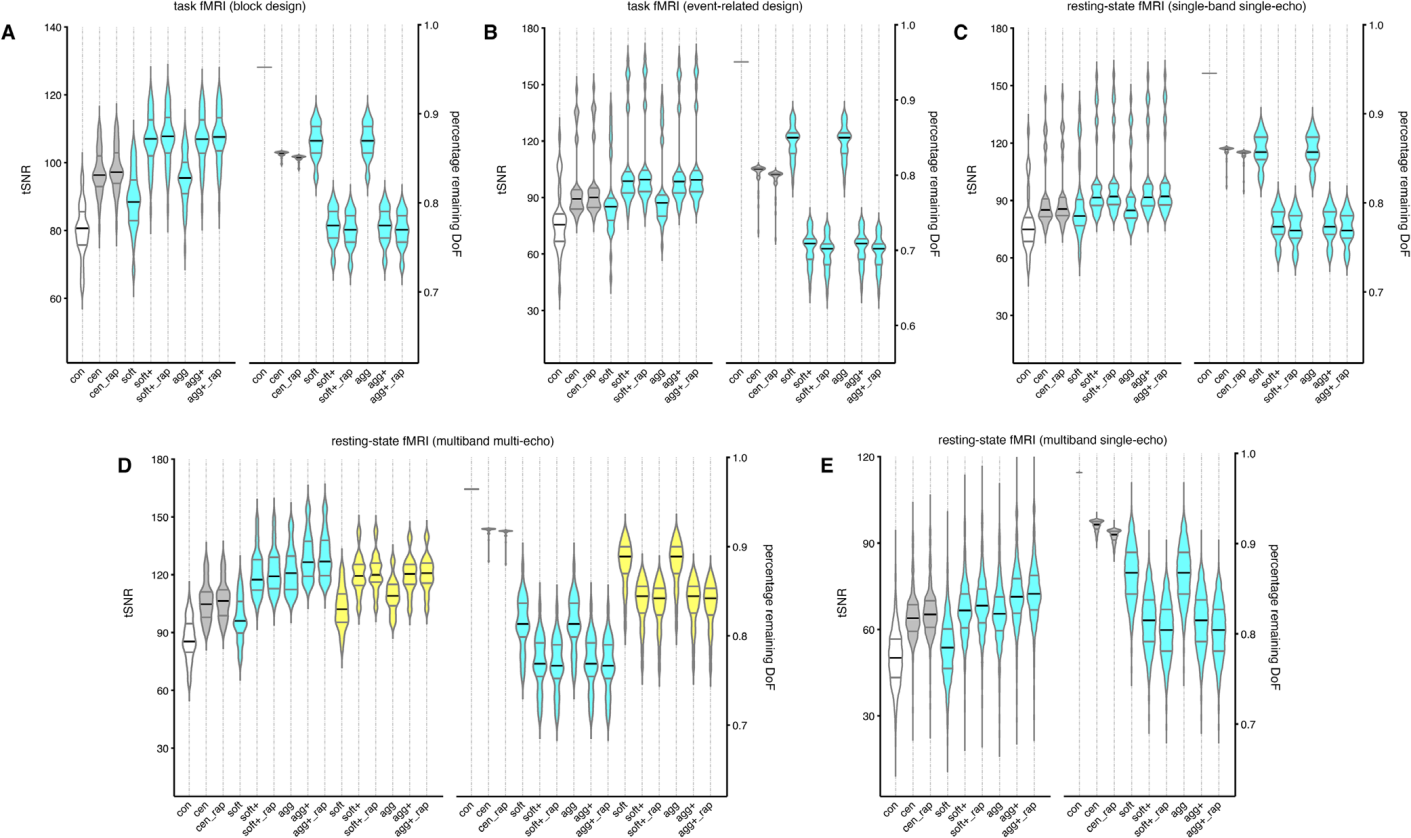
DoF‐corrected tSNR across all denoising pipelines for all 5 datasets. Violin plots of the tSNR (higher is better) and the percentage of remaining DoF (higher is better) across denoising pipelines for the block design n‐back task‐based fMRI (A); the event‐related design flanker task‐based fMRI (B); the single‐band single‐echo resting‐state fMRI (C); the multiband multi‐echo resting‐state fMRI (D); and the multiband single‐echo resting‐state fMRI (E). For the multi‐echo fMRI, violins filled with light cyan are the group of AROMA pipelines, and violins filled with light yellow are the group of tedana pipelines.

We calculated the tSNR to evaluate the overall data quality after denoising with each pipeline. As shown in Figure 1A–E, in all five datasets, tSNR increased as more denoising terms were added (e.g., soft+ vs. soft). When compared to the “con” pipeline, which included no denoising regressors, the time courses processed by all other pipelines showed higher whole‐brain average tSNRs. Overall, the ICA‐only pipelines (i.e., the “soft” and the “agg” for either the AROMA or the tedana related pipelines) performed slightly better in terms of tSNR than the control pipeline. Pipelines that incorporated head motion and aCompCor regressors (i.e., “cen”, “cen_rap”, “soft+”, “soft+_rap”, “agg+”, and “agg+_rap”) showed further improvement in tSNR compared to the ICA‐only pipelines. For the mbme‐rsfMRI dataset (Figure 1D), the multi‐echo ICA‐based tedana pipeline generally showed equivalent tSNR in the “soft” series pipelines (i.e., “soft”, “soft+”, “soft+_rap”), but lower tSNR in the “agg” series pipelines (i.e., “agg”, “agg+”, “agg+_rap”), when compared with AROMA ICA‐based pipelines. Among these, pipelines that included the additional rapidtide regressor showed slightly higher tSNR than their non‐rapidtide counterparts. In terms of DoF, pipelines with higher tSNR generally exhibited higher cost in DoF as well. The ICA‐plus pipelines (i.e., “soft+”, “agg+”, and their rapidtide counterparts) generally cost more DoF compared with the censoring pipelines. This difference was more pronounced in datasets with faster acquisitions (Figure 1D,E). The multi‐echo‐based tedana was able to retain much more DoF compared to the AROMA.

### 3.2 Effectiveness of Motion Correction

**FIGURE 2:**
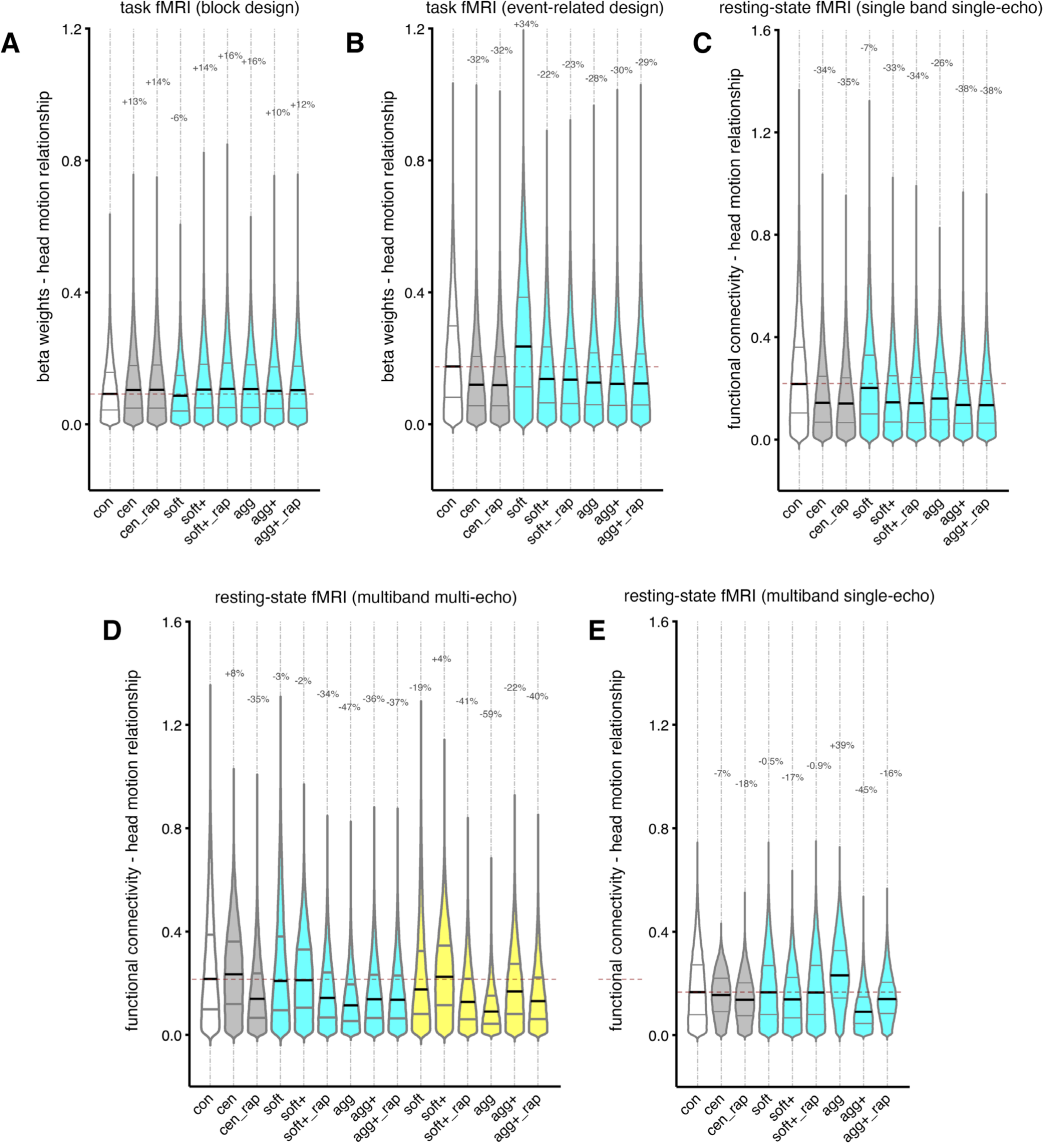
Effectiveness of motion correction. Violin plots of the relationship between the task‐based activation/resting‐state functional connectome and the mean head motion (lower is better) across denoising pipelines for the block design n‐back task (A); for the event‐related design flanker task (B); for the single‐band single‐echo resting‐state fMRI (C); for the multiband multi‐echo resting‐state fMRI (D); and for the multiband single‐echo resting‐state fMRI (E). For the multi‐echo fMRI, violins filled with light cyan are the group of AROMA pipelines, while violins filled with light yellow are the group of tedana pipelines. Red dotted lines represent the median level of the control pipeline for reference. The percentage number above each violin in the violin plots indicates the percentage increase/decrease of the median activation—head motion relationship/connectivity—head motion relationship of that pipeline relative to the control pipeline (con).

We calculated the task activation beta weights (for task‐based fMRI) and a functional connectome (for resting‐state fMRI) on the denoised time courses to assess the effectiveness of motion correction across all pipelines. Figure 2A,B shows the relationship between beta weights and the head motion for the n‐back and the flanker task‐based fMRI datasets. Figure 2C−E shows the functional connectome—head motion relationship for the sbse‐rsfMRI, mbme‐rsfMRI, and mbse‐rsfMRI datasets.

***bd‐tfMRI*:** For the block design n‐back task, the median correlation (absolute value of Fisher‐*Z* transformed *r* values) between activation and head motion for the control pipeline was 0.09, and the activation—head motion relationship did not fluctuate much across denoising pipelines, ranging from −6% (soft) to +16% (soft+_rap and agg) when compared to the median of the control pipeline (Figure 2A).

***er‐tfMRI*:** For the event‐related flanker task, the median activation—head motion correlation (absolute value of Fisher‐*Z* transformed *r* values) for the control pipeline was 0.18, and all denoising pipelines showed decreased activation—head motion relationship ranging from −32% (cen and cen_rap) to −22% (soft+), with the only exception of the AROMA soft pipeline, which showed a 34% increase in the activation—head motion relationship when compared to the control pipeline (Figure 2B).

***sbse‐rsfMRI*:** For the sbse‐rsfMRI, the median connectome—head motion correlation (absolute value of Fisher‐*Z* transformed *r* values) for the control pipeline was 0.22, and all denoising pipelines showed decreased connectome—head motion relationship ranging from −38% (agg+ and agg+_rap) to −7% (soft), compared to the control pipeline (Figure 2C).

***mbme‐rsfMRI*:** The connectome—head motion relationship for the mbme‐rsfMRI is shown in Figure 2D. The control pipeline showed median connectome—brain correlation (absolute value of Fisher‐*Z* transformed *r* values) at 0.22. The motion scrubbing pipeline “cen” showed slightly reduced motion correction with an 8% increase in the connectome—brain relationship. When paired with rapidtide, the censoring pipeline (cen_rap) showed improved motion correction with a 35% reduction in connectome—brain relationship compared to the control pipeline. In addition to the control pipeline (hollow) and the motion censoring pipelines (dark gray), the ICA‐involving pipelines are shown in two groups: the AROMA group (light cyan) and the tedana group (light yellow). Overall, the AROMA related pipelines outperformed the control pipelines with the decreased connectome—head motion relationship ranging from −47% (AROMA “agg”) to −2% (AROMA “soft+”). For the tedana related pipelines, all showed decreased connectome—head motion relationship compared to the control ranging from −59% (tedana “agg”) to −19% (tedana “soft”), with the tedana “soft+” pipeline as the only exception, which showed slightly increased (4%) connectome—head motion relationship compared to the control pipeline.

***mbse‐rsfMRI*:** For the mbse‐rsfMRI, the median connectome—head motion correlation (absolute value of Fisher‐*Z* transformed *r* values) for the control pipeline was 0.17, and all denoising pipelines showed decreased connectome—head motion relationship ranging from −45% (agg+) to −0.5% (soft), compared to the control pipeline, with the exception of the AROMA “agg” pipeline, which showed 39% increase in the connectome—head motion relationship (Figure 2E).

### 3.3 Effectiveness of Signal Preservation

After comparing the tSNR and the motion correction effectiveness that served as the mathematical upper and lower boundaries of the data quality, we then evaluated these pipelines empirically for their effectiveness of signal preservation. For our signals of interest, we chose the activation for task‐based fMRI and canonical functional connectivity for resting‐state fMRI.

**FIGURE 3:**
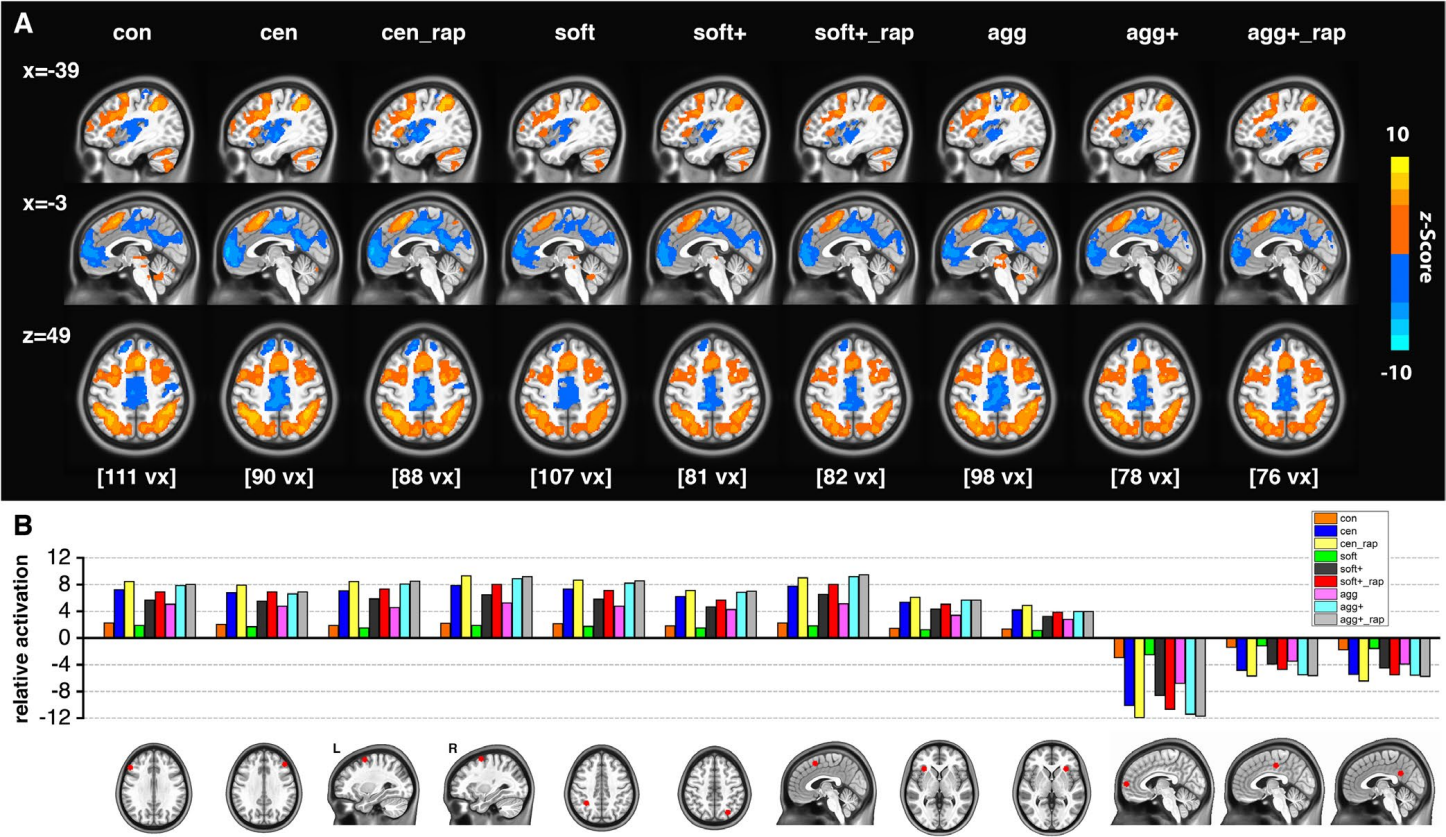
N‐back task activation pattern and relative activation of key ROIs. (A) The n‐back task activation pattern obtained from all denoising pipelines; the numbers in brackets denote the number of voxels needed for the cluster‐level multiple comparison correction (lower is better). (B) The relative activation of key ROIs (higher is better). ROI, region‐of‐interest.

***bd‐tfMRI*:** For the block design n‐back task, we used the working memory activation (2‐back vs. 0‐back contrast) as a demonstration. As shown in Figure 3A, the group‐level activation pattern of the n‐back task was visually similar across different pipelines (including the control pipeline). The number of voxels required for a cluster to survive the cluster‐based correction generally gets smaller as the number of denoising terms increases in the GLM. For the working memory activation pattern, 9 ROIs of activation and 3 ROIs of de‐activation were defined based on the mega‐mean map of the activation. The 9 activation regions included the left dorsolateral prefrontal cortex (dlPFC) [−51, 29, 31]; right dlPFC [45, 35, 33]; left frontal eye field (FEF) [−29, 3, 63]; right FEF [31, 1, 65]; left inferior parietal lobule (IPL) [−33, −49, 41]; right IPL [29, −69, 47]; dorsomedial prefrontal cortex (dmPFC) [−3, 13, 55]; left anterior insula (aIns) [−33, 25, 1]; and right aIns [31, 25, 1]. The three de‐activation regions included the ventromedial prefrontal cortex (vmPFC) [−3, 63, 7]; PCC (BA31d) [−3, −19, 45]; and PCC (BA23v) [−1, −49, 29]. The ROI‐specific relative activation of the n‐back task showed a clear distinction between pipelines. As shown in Figure 3B, the AROMA soft‐only pipeline (“soft”) performed relatively poorly, showing an average of 16% decrease across all key ROIs compared to the control pipeline. The pipelines “cen”, “soft+”, “agg”, and “agg+” performed better, with an average of 230%, 168%, 125% and 260% increased relative activation, respectively, compared to the control pipeline across all ROIs. The relative activation of the “cen_rap”, “soft+_rap”, and “agg+_rap” pipelines was boosted even further across all activated ROIs, with average increases of 285%, 226%, and 268% compared to the control pipeline.

The associations between the regional activation/de‐activation (2‐ vs. 0‐back contrast) of these ROIs and the behavioral performance of working memory d‐prime and reaction time (2‐back) were further evaluated and are shown in Figures S2 and S3. Among these activated/de‐activated regions, the bilateral FEF showed a significant positive correlation with the d‐prime, with correlation coefficients *r* ranging from 0.31 to 0.56. The vmPFC and PCC (BA31d) showed a significant negative correlation between their regional activation and the d‐prime, with correlation coefficients *r* ranging from −0.30 to −0.50 consistently across denoising pipelines. In contrast, the right FEF showed a significant negative correlation (with correlation coefficients *r* ranging from −0.40 to −0.51), and the PCC (BA31d) showed a significant positive correlation (with correlation coefficients *r* ranging from 0.32 to 0.40) between their regional activation and reaction time, consistently across denoising pipelines.

**FIGURE 4:**
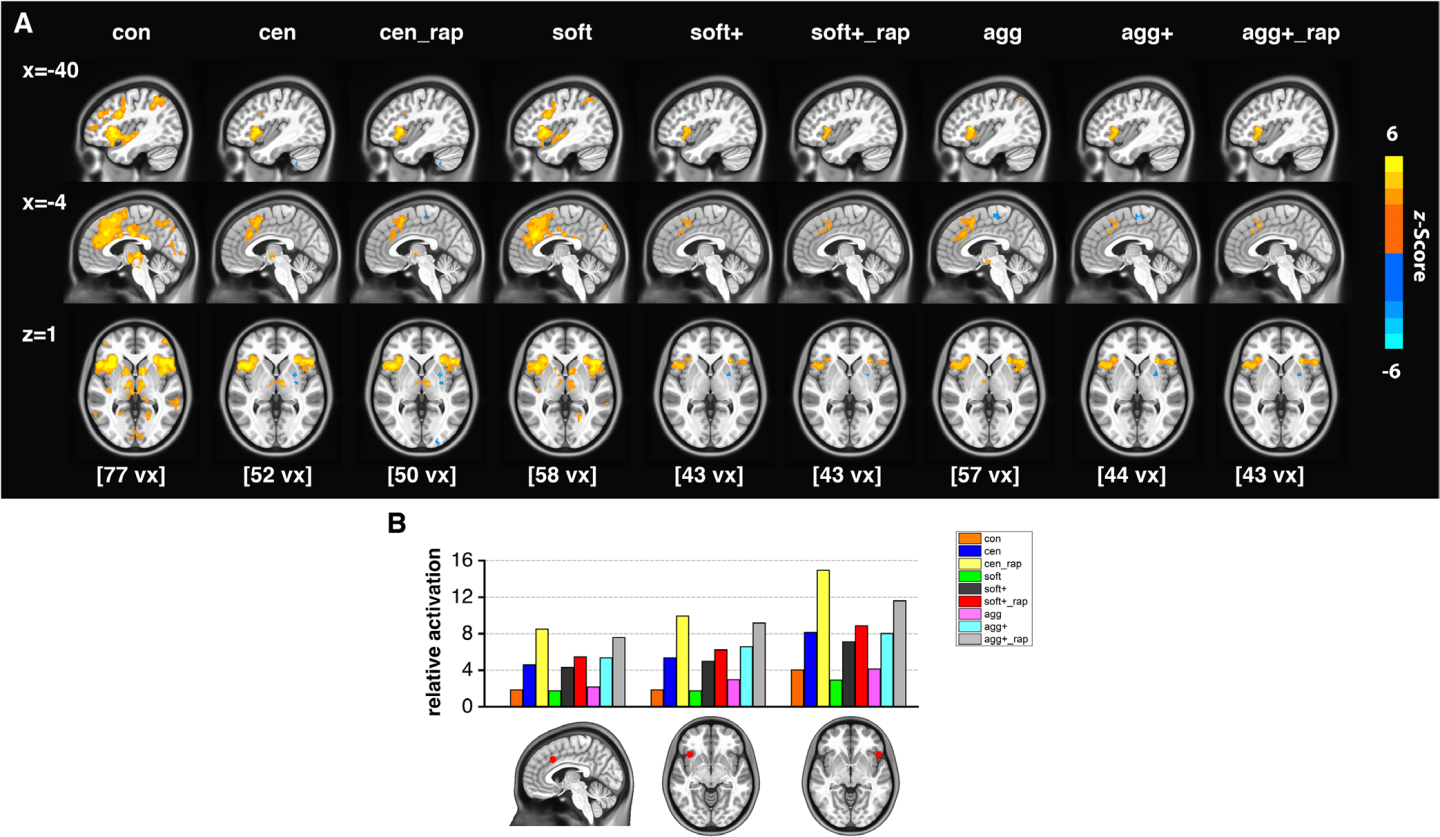
Flanker task activation pattern and relative activation of key ROIs. (A) The flanker task error‐related activation pattern obtained from all denoising pipelines; the numbers in brackets denote the number of voxels needed for the cluster‐level multiple comparison correction (lower is better). (B) The relative activation of key ROIs (higher is better). ROI, region‐of‐interest.

***er‐tfMRI*:** For the event‐related design flanker task‐based fMRI dataset, we used the error‐related activation (incorrect vs. correct contrast) as the metric. As shown in Figure 4A, the group‐level activation pattern of the flanker task varied across pipelines. Generally, all pipelines showed activation in the dorsal anterior cingulate cortex (dACC) and aIns bilaterally; however, the control pipeline and the AROMA soft only pipeline (“soft”) showed the activation pattern to a greater extent, especially in the dmPFC, middle cingulate cortex (MCC), dlPFC, IPL, and thalamus. For the flanker task error‐related activation pattern, three ROIs were defined from the mega‐mean map: dACC [5, 21, 37], left aIns [−37, 21, −7], and right aIns [49, 21, −5]. The ROI‐specific relative activation of the flanker task exhibited a clear distinction between pipelines. As shown in Figure 4B, the AROMA‐only pipelines (“soft” and “agg”) performed poorly in relative activation, with an average of 13% decrease and 25% increase compared to the control pipeline. The pipelines “cen”, “soft+”, and “agg+” performed much better, with an average of 142%, 122%, and 175% increase in relative activation respectively, compared to the control pipeline across all ROIs. The relative activation of the “cen_rap”, “soft+_rap”, and “agg+_rap” pipelines consistently showed improved performance across all activated ROIs, with an average increase of 344%, 178%, and 287% respectively compared to the control pipeline.

**FIGURE 5:**
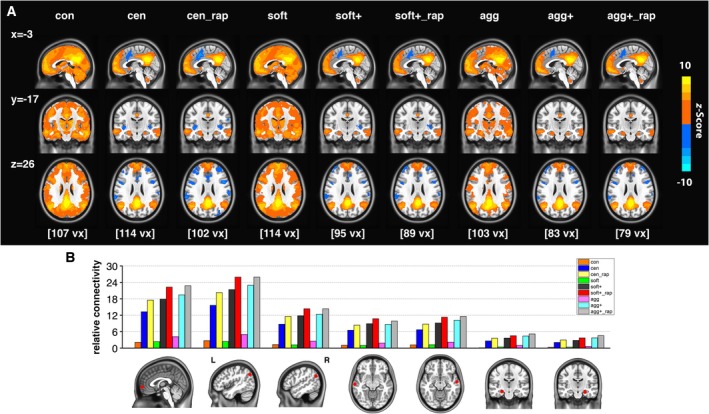
sbse‐rsfMRI functional connectivity pattern (PCC) and relative connectivity of key ROIs. (A) The PCC functional connectivity pattern obtained from all denoising pipelines. The numbers in brackets denote the number of voxels needed for the cluster‐level multiple comparison correction (lower is better). (B) The relative connectivity of key ROIs (higher is better). ROI, region‐of‐interest; PCC, posterior cingulate cortex.

***sbse‐rsfMRI*:** For resting‐state fMRI datasets, we used the PCC functional connectivity for demonstration. The results of the sbse‐rsfMRI are shown in Figure 5A. The control pipeline and the AROMA soft only pipeline (“soft”) performed poorly, exhibiting a non‐specific enhancement of the connectivity pattern that nearly resembles a whole‐brain pattern. The AROMA aggressive‐only pipeline (“agg”) outperformed the “con” and “soft” pipelines, but still showed a spatially extended pattern with sharp edges formed across scattered (but connected) voxels. The “cen”, “soft+”, “agg+”, as well as their rapidtide counterparts all showed spatial patterns smoothly resembling the DMN topology. For the PCC functional connectivity pattern, 7 ROIs were defined from the mega‐mean map: vmPFC [−1, 61, −5], left angular gyrus (AG) [−45, −71, 37], right AG [51, −67, 31], left middle temporal gyrus (MTG) [−63, −17, −11], right MTG [59, −9, −13], left hippocampus [−25, −19, −17], and right hippocampus [25, −17, −19]. The ROI‐specific relative connectivity of the PCC also exhibited a clear distinction between pipelines. As shown in Figure 5B, the AROMA‐only pipelines (“soft” and “agg”) performed poorly in relative connectivity, with an average of 20% and 125% relative increase compared to the control pipeline. The pipelines “cen”, “soft+”, and “agg+” performed much better, with an average of 560%, 808%, and 923% increased relative connectivity value respectively compared to the control pipeline across all ROIs. The relative connectivity of the “cen_rap”, “soft+_rap”, and “agg+_rap” pipelines consistently increased across all activated ROIs, with average gains of 789%, 1021%, and 1087% respectively compared to the control pipeline.

**FIGURE 6:**
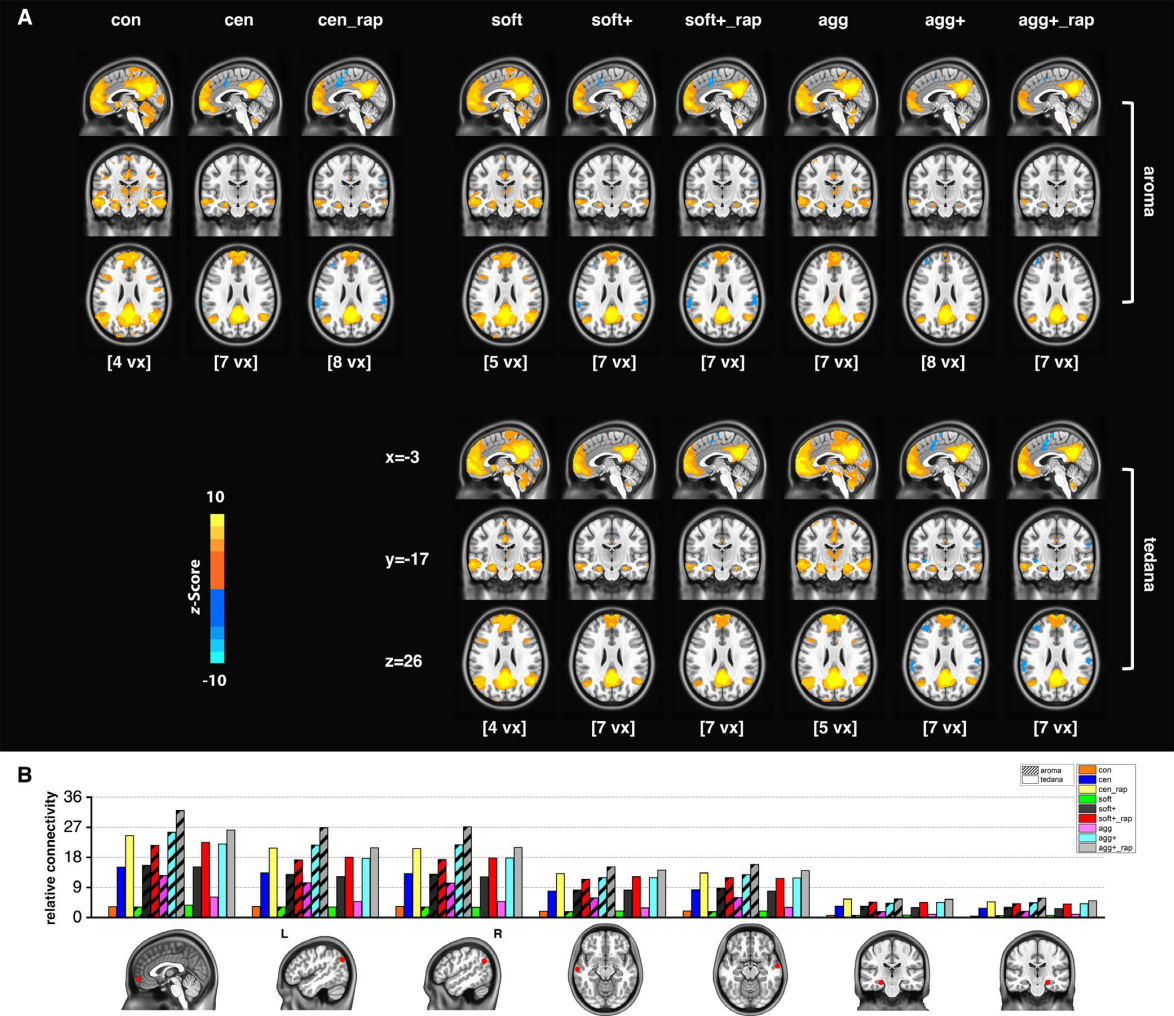
mbme‐rsfMRI functional connectivity pattern (PCC) and relative connectivity of key ROIs. (A) The PCC functional connectivity pattern obtained from all denoising pipelines. The numbers in brackets denote the number of voxels needed for the cluster‐level multiple comparison correction (lower is better). (B) The relative connectivity of key ROIs (higher is better). ROI, region‐of‐interest; PCC, posterior cingulate cortex.

***mbme‐rsfMRI*:** For the mbme‐rsfMRI, as shown in Figure 6A, the control pipeline and the soft only pipelines (“soft”) for “AROMA,” “tedana,” the aggressive‐only pipeline (“agg”) for AROMA, and tedana were able to roughly show the canonical DMN pattern, but also showed a spatially extended noisy pattern with sharp edges formed with scattered but connected voxels. The “cen”, “soft+”, and “agg+” for both AROMA and tedana pipelines, as well as their rapidtide counterparts, all showed a reasonable pattern restricted to the DMN topology, which also included small brain structures such as the hippocampus. For the multi‐echo resting‐state PCC functional connectivity pattern, 7 ROIs were defined from the mega‐mean map: vmPFC [−1, −59, −9], left AG [−47, −73, 35], right AG [51, −65, 31], left MTG [−63, −15, −13], right MTG [61, −7, −15], left hippocampus [−25, −23, −15], and right hippocampus [23, −21, −15]. The ROI‐specific relative connectivity of the PCC also exhibited clear differences between pipelines. As shown in Figure 6B, the ICA‐only (AROMA/tedana, “soft”/“agg”) pipelines performed poorly in relative connectivity, with an average of 1% decrease and 8% increase for the “soft” pipelines of AROMA and tedana respectively compared to the control pipeline and average increases of 208% and 59% respectively for the “agg” pipelines of AROMA and tedana. Overall, the scrubbing pipelines (“cen” and “cen_rap”), and the “plus” variants for both AROMA and tedana (“soft+”, “soft+_rap”, “agg+”, and “agg+_rap”) performed reasonably well, with increases in relative connectivity respectively ranging from 306% to 725% compared to the control pipeline. Including the rapidtide regressor in the denoising pipelines was beneficial in this multi‐echo dataset and shows substantially improved relative connectivity for the “cen_rap”, “soft+_rap”(AROMA), “soft+_rap” (tedana), “agg+_rap” (AROMA), and “agg+_rap” (tedana), with respective increases in relative connectivity of 581%, 490%, 501%, 725%, and 610% compared to the control pipeline.

**FIGURE 7:**
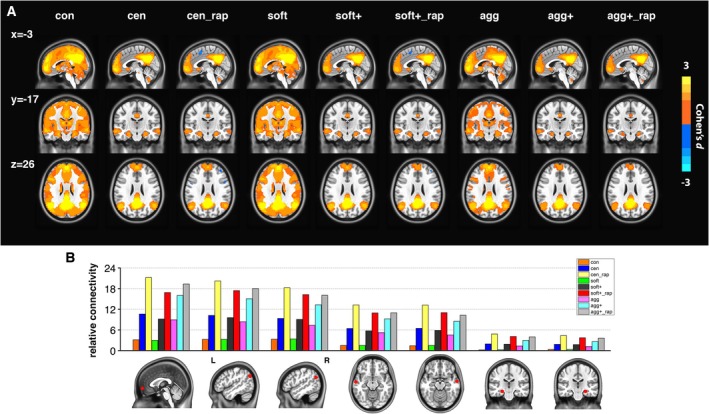
mbse‐rsfMRI functional connectivity pattern (PCC) and relative connectivity of key ROIs. (A) The PCC functional connectivity pattern obtained from all denoising pipelines. (B) The relative connectivity of key ROIs (higher is better). ROI, region‐of‐interest; PCC, posterior cingulate cortex.

***mbse‐rsfMRI*:** For the mbse‐rsfMRI dataset, as shown in Figure 7A, the control pipeline and the AROMA soft only pipeline (“soft”) performed poorly, exhibiting a non‐specific enhancement of the connectivity pattern that nearly resembles a whole‐brain pattern. The AROMA aggressive‐only pipeline (“agg”) outperformed the “con” and “soft” pipelines, but still showed a spatially extended pattern with sharp edges formed with scattered (but connected) voxels. The “cen”, “soft+”, and “agg+”, as well as their rapidtide counterparts, all showed spatial patterns smoothly resembling the DMN topology. For the PCC functional connectivity pattern, 7 ROIs were defined from the mega‐mean map: vmPFC [0, 60, −8], AG [−48, −72, 35], right AG [50, −67, 30], left MTG [−60, −10, −13], right MTG [60, −7, −16], left hippocampus [−24, −22, −16], and right hippocampus [26, −22, −16]. The ROI‐specific relative connectivity of the PCC also exhibited a clear distinction between pipelines. As shown in Figure 7B, the AROMA soft pipelines (“soft”) performed poorly in relative connectivity, with an average increase of 4% compared to the control pipeline. The pipelines “cen”, “soft+”, “agg”, and “agg+” performed much better, with an average of 319%, 285%, 215%, and 504% increased relative connectivity value respectively compared to the control pipeline across all ROIs. The relative connectivity of the “cen_rap”, “soft+_rap”, and “agg+_rap” pipelines consistently increased across all activated ROIs, with average gains of 806%, 665%, and 660%, respectively compared to the control pipeline.

### 3.4 Additional Control Analyses

In addition to comparing pipelines on these indices for the three resting‐state datasets using the high‐pass temporal filtering in our primary results, we also provide results using the conventional band‐pass filtering between 0.01 and 0.1 Hz. However, when conducting the GLM analysis with band‐pass filtering regressors together with nuisance regression in a 1‐step manner (Caballero‐Gaudes and Reynolds 2017), only the sbse‐rsfMRI dataset (TR = 2 s) was able to remain rank sufficient (Figure S2). The regression matrix for the mbme‐rsfMRI dataset (TR = 1.33 s) became rank deficient for some of the high DoF cost pipelines (e.g., AROMA soft+, Figure S5B). This was more pronounced in the mbse‐rsfMRI dataset (TR = 0.8 s) (Figure S6B). Therefore, for these two datasets, we used a sequential approach in which band‐pass filtering was applied prior to nuisance regression, with the same filter applied to both the imaging data and regressors. This approach corresponds to the sequential orthogonalizing regressors in sequential regression as an alternative to the 1‐step regression (Lindquist et al. 2019). However, the group‐level rsFC patterns showed markedly reduced sensitivity for pipelines with low effective DoF (regardless of separating the bandpass filter into a separate procedural step) (Figures S5A and S6A). To further investigate this, we conducted additional analyses in which we varied the upper cutoff frequency of the band‐pass filter and compared functional connectivity patterns.

We demonstrated this with the mbme‐rsfMRI dataset using the “soft+” pipeline which suffered severely from rank deficiency at the 0.1 Hz cutoff. We first identified a “critical cutoff frequency” (0.11 Hz in our dataset); above this critical cutoff the design matrix was rank‐sufficient for all subjects, while below this the design matrix became rank‐deficient for some subjects. At and above this cutoff, PCC rsFC patterns were reliably recovered. Below this cutoff (e.g., 0.1 Hz), rsFC estimates were substantially degraded. Moreover, across a range of cutoff frequencies between 0.1 Hz and the Nyquist frequency, we observed a gradual restoration of expected rsFC patterns as the effective DoF/rank of matrix increased (Figure S7). We conducted two additional control analyses to rule out the possibility that what we observed was due to the difference between the 1‐step regression versus sequential filtering and GLM. We performed a bandpass version of the “soft+” pipeline for the sbse‐rsfMRI dataset, and a high‐pass version of the “soft+” pipeline for the mbme‐rsfMRI dataset; both were conducted in a two‐step sequential manner and compared with the corresponding 1‐step results. Both tests showed equivalent rsFC patterns between the 1‐step regression and two‐step sequential method (Figure S8).

To assess the effect of the censoring threshold, we also conducted an additional analysis using a more stringent frame‐to‐frame movement Enorm threshold (0.2 mm) in one representative cohort, the mbse‐fMRI dataset (Figure S10). We observed that: 1. the relative performance and ranking of pipelines remained largely unchanged compared to the main results (Figures 1E, 2E and 7), and 2. the rsFC patterns were qualitatively similar across both motion thresholds (Figures 7, S10).

## 4 Discussion

In our current study, we systematically evaluated fMRI denoising pipelines incorporating different combinations of nuisance regressors, including commonly used factors like head motion and physiological nuisance (e.g., TR censoring, head motion parameters, aCompCor, AROMA), as well as more recent methodological advances (e.g., multi‐echo denoising and modeling for time‐shifted physiological noise). All pipelines underwent GLM‐based denoising using a 1‐step regression approach, with temporal autocorrelation (pre‐whitening) modeled. We benchmarked these pipelines using several measures, including tSNR and percentage of remaining DoF, effectiveness of motion correction, and signal preservation across pipelines and across a range of datasets including single‐ and multi‐band, single‐ and multi‐echo acquisitions, as well as both resting‐state and task‐based fMRI. Our analyses revealed the following key findings:

**1. ICA‐only (soft/aggressive) is not sufficient**. Our results demonstrated that ICA‐based denoising alone (i.e., AROMA soft/agg, tedana soft/agg) is not sufficient for optimal fMRI denoising. These pipelines exhibited poor performance in several aspects, including ineffective motion correction and reduced specificity of both task activation (e.g., in the flanker task) and rsFC patterns, particularly in the single‐echo dataset. Further, these pipelines demonstrated low specificity in capturing task‐induced activation and/or rsFC in key brain regions, as reflected by lower relative activation or functional connectivity of the key node ROIs relative to the whole‐brain averages. Users should be cautious when considering using derivative outputs from these software packages named “xxx‐cleaned” or “xxx‐denoised,” as these derivatives are denoised only on the noise components that the software package was specifically intended for. These outputs are better used along with additional denoising procedures (e.g., aCompCor, rapidtide, etc.) as suggested by the current findings. It is more desirable to obtain the time series of the components that are identified as “noise” components from those derivative directories, and use them together with other noise regressors (as well as temporal filter regressors) to form a full regression matrix and denoise in a single step GLM (i.e., the 1‐step regression framework as we demonstrated in our benchmarking).

**2. Supplement of physiological confounds greatly enhances the efficacy of denoising**. Among the denoising pipelines we tested, the “cen”, “soft+” (both AROMA and/or tedana), and “agg+” (both AROMA and/or tedana) pipelines incorporate additional confounds representing physiological noise (aCompCor) into the GLM, alongside head motion‐related nuisance regressors. These pipelines outperformed the control pipeline in denoising, demonstrating higher overall tSNR, weaker correlations between motion and task activation or functional connectivity, and stronger task‐induced activation and/or functional connectivity in key regions. Of note, although regression‐based denoising (such as AROMA and tedana) tends to be less effective against burst‐type noise (Mehta et al. 2024), we observed that the censoring‐based and ICA‐based pipelines performed comparably when physiological confounds were included (aCompCor). From a practical standpoint, the censoring‐based pipelines are simpler to implement, as no ICA decomposition or signal‐noise classification is needed. In contrast, the ICA‐based pipelines show an advantage in preserving temporal continuity, highlighting a tradeoff between implementation complexity and potential benefits.

**3. Modeling physiological confounds with temporal lag improves denoising even more**. Including the rapidtide regressor in the GLM pipelines further improved denoising performance across nearly all benchmarks, with particularly pronounced effects observed in the mbme‐rsfMRI and mbse‐rsfMRI datasets. These improvements could potentially be attributed to the fast acquisition allowed in these multiband scanning protocols that provided richer temporal information. This also could be partially attributed to the benefits of multi‐echo data in extracting time‐lagged correlations in physiological noise. Specifically, the “optimally combined” data from multiple echoes provides an intrinsically higher SNR, allowing more accurate modeling of time‐lagged correlations for physiological confounds.

**4. Degrees‐of‐freedom (DoF) cost and implications for temporal filtering**. DoF are a significant factor in high‐dimensional time series data such as fMRI. Retaining more DoF results in a more stable and reliable calculation of the inverse of the regression matrix, which improves the accuracy of parameter estimates and their associated statistical inference in GLM. Therefore, given the same denoising performance, pipelines with less DoF cost (more remaining DoF) are preferable. Among the pipelines we tested, the augmented ICA pipelines (i.e., “soft+”, “soft+_rap”, “agg+”, “agg+_rap”) generally incurred greater DoF costs than the censoring‐based/scrubbing pipelines (i.e., “cen”, “cen_rap”). However, this may differ between datasets due to factors including original data quality and head motion. Another relevant aspect of DoF consumption in fMRI (particularly rs‐fMRI) is temporal band‐pass filtering. Consistent with our emphasis on 1‐step regression in GLM denoising, it is recommended to include all nuisance regressors (including the temporal filter) in the regression matrix, to properly account for the DoF cost in GLM‐based denoising (Caballero‐Gaudes and Reynolds 2017; Hallquist et al. 2013).

Band‐pass filtering has long been used in rs‐fMRI data to retain low‐frequency fluctuations roughly ranging from 0.01 to 0.1 Hz (Fox and Raichle 2007; Fransson 2005), while suppressing physiological noise such as respiratory (~0.2 Hz) and cardiac confounds (~1–1.2 Hz) (Behzadi et al. 2007; Tong et al. 2019). However, this approach is becoming less optimal for modern data analysis for several reasons: 1. These noise components may not be fully sampled, especially their harmonics, but rather aliased into lower frequencies, rendering the band‐pass strategy less effective to begin with (although mitigated to some degree with faster acquisition); 2. With the advancement of analytical techniques, there are perhaps better ways to address the physiological noise (e.g., aCompCor, polynomial detrending, multi‐echo acquisition and denoising, rapidtide etc.); 3. Referring to fluctuation within this frequency band as “noise” itself is questionable as it has been demonstrated that the spontaneous neuronal activity might contain higher frequency components, even up to 0.8 Hz (Boubela et al. 2013; Chen and Glover 2015; Lee et al. 2013). Therefore, band‐pass filtering could potentially remove signals of interest at higher frequency bands (Griffanti et al. 2014; Pruim, Mennes, van Rooij, et al. 2015). Examination of the power spectral density of the denoised time series in our datasets showed that, although power was generally attenuated, substantial power still remained near the upper cutoff frequency of 0.1 Hz, even for the most aggressive denoising pipelines (Figure S9); and 4. Practically, including band‐pass regressors can incur a significant DoF cost. Band‐pass filtering at 0.01–0.1 Hz costs ~63% of the total DoF in the sbse‐rsfMRI dataset (TR = 2 s), same filter costs ~75% of the total DoF in the mbme‐rsfMRI dataset (TR = 1.33 s), and ~85% of the total DoF in the mbse‐rsfMRI dataset (TR = 0.8 s). This figure will increase and become even more problematic as the temporal resolution of the fMRI data increases. For a given scan duration at this band‐pass range, the usable DoF within the pass band remains constant for any TR; lowering the TR simply increases the higher frequency data, see (Caballero‐Gaudes and Reynolds 2017) for a discussion. Additionally, while scans with shorter TRs are associated with higher percentage of DoF cost in band‐pass, the number of nuisance regressors estimated also tend to be much higher (Figures S5 and S6), as they increase with the number of time points.

These factors collectively impact the rank sufficiency or effective DoF of the GLM regression matrix, which cannot be resolved by conducting temporal filtering and the nuisance regression in sequential steps (if effective DoF are not sufficient to begin with). In our current study, we applied the high‐pass filter to our fMRI data and showed effective denoising performance and preserved spatial patterns consistent with the traditional understanding of the n‐back task activation, error‐related activation, and DMN functional connectivity. The rsFC patterns are also highly similar to the band‐pass version of the results (Figure S2), without incurring a significant DoF cost. Based on these findings, we suggest that future studies using modern acquisition protocols consider omitting band‐pass filtering, which is negatively impacted by factors associated with these modern protocols such as faster acquisition. Instead, we suggest using high‐pass temporal filtering combined with other denoising techniques that could benefit from these technical advancements such as multi‐echo denoising, aCompCor, and rapidtide.

### 4.1 Additional Notes

In our study, we used the Enorm of 0.35 mm as the frame‐to‐frame censoring threshold. This is a pragmatic balance between censoring high‐motion volumes and retaining sufficient data/sample, particularly in clinical cohorts where sample sizes are often limited and motion tends to be higher. This threshold falls within commonly used ranges in fMRI studies (Ciric et al. 2017; Power et al. 2012; Satterthwaite et al. 2013) and has been applied in prior works in clinical study applications (Biernacki et al. 2026; Liang et al. 2015; Zhai et al. 2023). Although the primary goal of this study is to compare denoising pipelines rather than to optimize censoring threshold, shorter TRs in modern acquisition may warrant more stringent thresholds that may influence data quality. Therefore, we also conducted an additional analysis using a more stringent Enorm threshold of 0.2 mm in one representative cohort, the mbse‐fMRI dataset. While overall performance metrics increased under the more stringent threshold, this improvement was not specific to the censoring‐related pipelines, but was observed across all pipelines and is likely driven by the effect of sample selection. As a contrast, the more stringent threshold resulted in a substantial reduction in usable sample size (from *N* = 847 to *N* = 624), reflecting the exclusion of higher‐motion subjects. This highlights an important practical tradeoff: more stringent censoring could potentially improve data quality but can substantially reduce sample size, which is often a critical factor in clinical studies.

The index *Effectiveness of motion correction* that we benchmarked showed relatively less consistency across different datasets in pipeline benchmarking. This index is conceptually similar to the QC‐FC index that has been frequently used in benchmarking studies (Ciric et al. 2017; Parkes et al. 2018). However, the basic assumption of this index that better denoising should show no true relationship between motion and functional connectivity has been demonstrated as potentially flawed. Emerging evidence has shown a true association between head motion and functional connectivity/task activations (Bolton et al. 2020; Williams et al. 2022; Zeng et al. 2014). Our observed inconsistency in this index between datasets could potentially be attributed to this issue. Additional benchmarking indices such as the mean absolute change in resting state functional connectivity (MAC‐RSFC) or the delta mean squared error resting state functional connectivity (ΔMSE‐RSFC) (Williams et al. 2022) should be explored in future pipeline benchmarking studies.

## 5 Conclusion

Our results demonstrated the effectiveness of various well‐established noise regressor sets in a 1‐step GLM manner, revealing a spectrum of performance from insufficient (“soft”, “agg”) to effective (“cen”, “soft+”, “agg+”, and their rapidtide “_rap” variants). Based on our findings, we draw the following conclusions: First, censoring‐based pipelines (“cen”, “cen_rap”) and ICA+ pipelines (“soft+”, “soft+_rap”, “agg+”, “agg+_rap”) generally performed equivalently well in denoising. Second, augmenting pipelines with rapidtide regressor (“_rap”) enhances denoising performance, especially for multiband and/or multi‐echo resting‐state data. Third, high‐pass filtering shows effective denoising without the huge DoF cost associated with band‐pass filtering, and is thus recommended when combined with other advanced denoising techniques (e.g., tedana, aCompCor, rapidtide), especially for fMRI dataset with higher temporal resolutions.

Taken together, our study provides a comprehensive evaluation of standard denoising techniques across a range of fMRI designs (resting‐state, block design, and event‐related tasks) and acquisition protocols (single‐ and multi‐band, single‐ and multi‐echo) in a 1‐step regression framework. These results offer valuable insights for researchers to make informed decisions about which denoising procedures to apply based on their specific dataset. Our study also provides a descriptive approach to benchmarking and suggests ongoing benchmarking to ensure the continued advancement of fMRI data processing as new techniques or new applications of existing techniques emerge.

## Author Contributions

Conceptualization: T.Z., T.J.R., Y.Y. and A.C.J. Data curation: T.Z. Formal analysis: T.Z. Funding acquisition: Y.Y. and A.C.J. Investigation: T.Z. and H.G. Methodology: T.Z., H.G., B.B.F. and T.J.R. Project administration: A.C.J. Software: T.Z. Resources: Y.Y. and A.C.J. Supervision: A.C.J. Validation: T.Z. Visualization: T.Z., A.H. and E.C. Writing – original draft: T.Z. Writing – review and editing: T.Z., H.G., A.H., E.C., B.B.F., T.J.R., Y.Y. and A.C.J.

## Funding

This work was supported by the National Institute on Drug Abuse Intramural Research Program, NIH, ZIA DA000641.

## Conflicts of Interest

The authors declare no conflicts of interest.

## Supporting information


**Supplementary Method:** Task design for 2 task fMRI datasets.
**Figure S1:** whole brain 411‐ROI atlas in the MNI space.
**Figure S2:** benchmarking across all denoising pipelines for the single‐band single‐echo resting‐state datasets (band‐pass version).
**Figure S3:** relationship between working memory activation and task performance (d‐prime) of key‐ROIs across pipelines.
**Figure S4:** relationship between working memory activation and task performance (reaction time) of key‐ROIs across pipelines.
**Figure S5:** multiband multi‐echo resting‐state fMRI functional connectivity pattern (PCC) (band‐pass version).
**Figure S6:** multiband single‐echo resting‐state fMRI functional connectivity pattern (PCC) (band‐pass version).
**Figure S7:** Effective DoF affects reliable estimation of functional connectivity pattern.
**Figure S8:** control analyses.
**Figure S9:** power spectral density of the denoised time series of the 3 resting‐state datasets for all compared pipelines.
**Figure S10:** benchmarking across all denoising pipelines for the multiband single‐echo resting‐state datasets using an alternative, more stringent Enorm threshold (0.2 mm).

**Suppoting Material:** mmpPlus_411_mni.zip

## Data Availability

The data used in this study are subject to the following licenses/restrictions: Raw data of Cohort A, B, and C, as well as codes and derived data supporting the findings of this study, are available from the corresponding author upon reasonable request and contingent on institutional approval. Raw data of Cohort D are from the publicly available Adolescent Brain Cognitive Development (ABCD) dataset.
