# Supplementary material for: Benchmarking fMRI Denoising Pipelines
Source: Hum Brain Mapp. 2026 Jul 1;47(10):e70561. doi: 10.1002/hbm.70561 (PMC13320824; doi:10.1002/hbm.70561)
Supplement: Supplementary file 1 — Supplementary Method: Task design for 2 task fMRI datasets. Figure S1: whole brain 411‐ROI atlas in the MNI space. Figure S2: benchmarking across all denoising pipelines for the single‐band single‐echo resting‐state datasets (band‐pass version). Figure S3: relationship between working memory activation and task performance (d‐prime) of key‐ROIs across pipelines. Figure S4: relationship between working memory activation and task performance (reaction time) of key‐ROIs across pipelines. Figure S5: multiband multi‐echo resting‐state fMRI functional connectivity pattern (PCC) (band‐pass version). Figure S6: multiband single‐echo resting‐state fMRI functional connectivity pattern (PCC) (band‐pass version). Figure S7: Effective DoF affects reliable estimation of functional connectivity pattern. Figure S8: control analyses. Figure S9: power spectral density of the denoised time series of the 3 resting‐state datasets for all compared pipelines. Figure S10: benchmarking across all denoising pipelines for the multiband single‐echo resting‐state datasets using an alternative, more stringent Enorm threshold (0.2 mm). [file HBM-47-e70561-s002.pdf]

Supplementary materials for:

Benchmarking fMRI Denoising Pipelines

Tianye Zhai, Ph.D.; Hong Gu, Ph.D.; Anika Holton, B.S.; Elanor Chang, B.S.; Blaise B. Frederick, Ph.D.; Thomas J. Ross, Ph.D.; Yihong Yang, Ph.D.; Amy C. Janes, Ph.D.

Content list:

Supplementary method: Task design for 2 task fMRI datasets

Fig.S1 whole brain 411-ROI atlas in the MNI space

Fig.S2 benchmarking across all denoising pipelines for the single-band single-echo resting-state datasets (band-pass version)

Fig.S3 relationship between working memory activation and task performance (d-prime) of key-ROIs across pipelines

Fig.S4 relationship between working memory activation and task performance (reaction time) of key-ROIs across pipelines

Fig.S5 multiband multi-echo resting-state fMRI functional connectivity pattern (PCC) (band-pass version)

Fig.S6 multiband single-echo resting-state fMRI functional connectivity pattern (PCC) (band-pass version)

Fig.S7 Effective DoF affects reliable estimation of functional connectivity pattern

Fig.S8 control analysis

Fig.S9 power spectral density of the denoised time series of the 3 resting-state datasets for all compared pipelines

Fig.S10 benchmarking across all denoising pipelines for the multiband single-echo resting-state datasets using an alternative, more stringent Enorm threshold (0.2 mm).

Supplementary Method: Task design for 2 task fMRI datasets

Participants in cohort A completed a 3-run (7min 4s / run) block design visual n-back working memory task during the fMRI scan. Each run started with 8s fixation and followed with two repetitions of four task blocks. Each repetition started with a 0-back block and 3 blocks of experiment conditions (1-, 2-, and 3-back) followed in a randomized order. Each block consisted of a 2-second short instruction indicating the current block type, a 30-second stimulus presentation of 15 trials, followed by a 20-second fixation. Each trial is 2000ms in length with the first 500ms displaying the visual stimulus. In each trial of the 1-, 2-, and 3-back blocks, a letter is presented in the center of the screen, and the participants were required to press a key if the current letter was the same as the letter displayed n trials before (n = 1, 2, 3). The 0-back served as the control condition and participants were instructed to respond to the pre-defined letter "D"/"d".

Participants in cohort B completed a 5-run (4min 48s / run) event-related parametric flanker task during the fMRI scan (Forster et al., 2011). The visual stimuli of the task consisted of an array of 7 letters ("H" or "S"), with the center letter being the target and the six remaining letters bilateral to the center target being the flankers. There were two event types in all stimuli: congruent, where flankers are the same as target ("HHHHHHH" or "SSSSSSS"); and incongruent, where flankers deviate from the target. For the incongruent trials, 3 levels of conflict were modeled in the task design: low-conflict: only the outermost flankers deviate from the target ("SHHHHHS" or "HSSSSSH"); medium-conflict: 2 flankers deviate from the target ("SSHHHSS" or "HHSSSHH"); and high-conflict: all flankers deviate from the target ("SSSHSSS" or "HHHSHHH"). There were 240 trials in total, 120 being congruent and 120 incongruent (40 trials each conflict level). Each trial starts with a fixation followed by 100ms of flanker display, then the whole flankers and target display for an additional 750ms. The ITI was randomly jittered from 500ms to 7000ms. Participants were instructed to respond with accuracy and reaction time equally prioritized.

Fig.S1 whole brain 411-ROI atlas in the MNI space

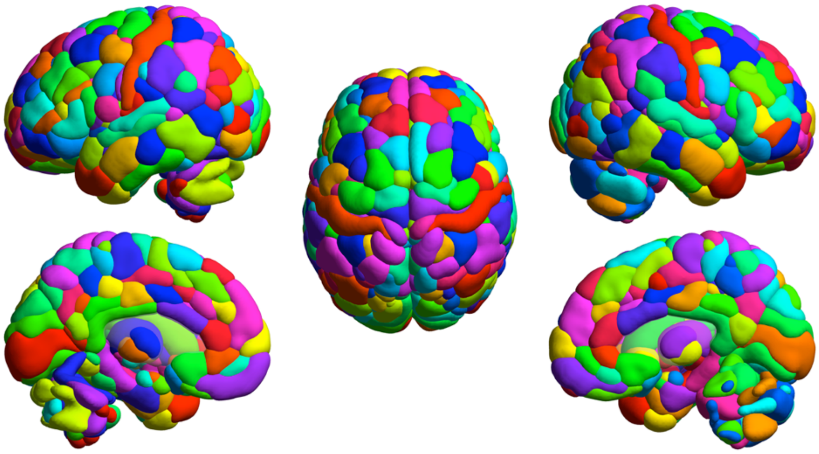

The 411-ROI volumetric cortical and subcortical atlas in the MNI space (available as "mmpPlus_411_mni.zip").

Abbreviations: ROI, region-of-interest; MNI, the Montreal Neurological Institute.

Fig.S2 benchmarking across all denoising pipelines for the single-band single-echo resting-state datasets (band-pass version)

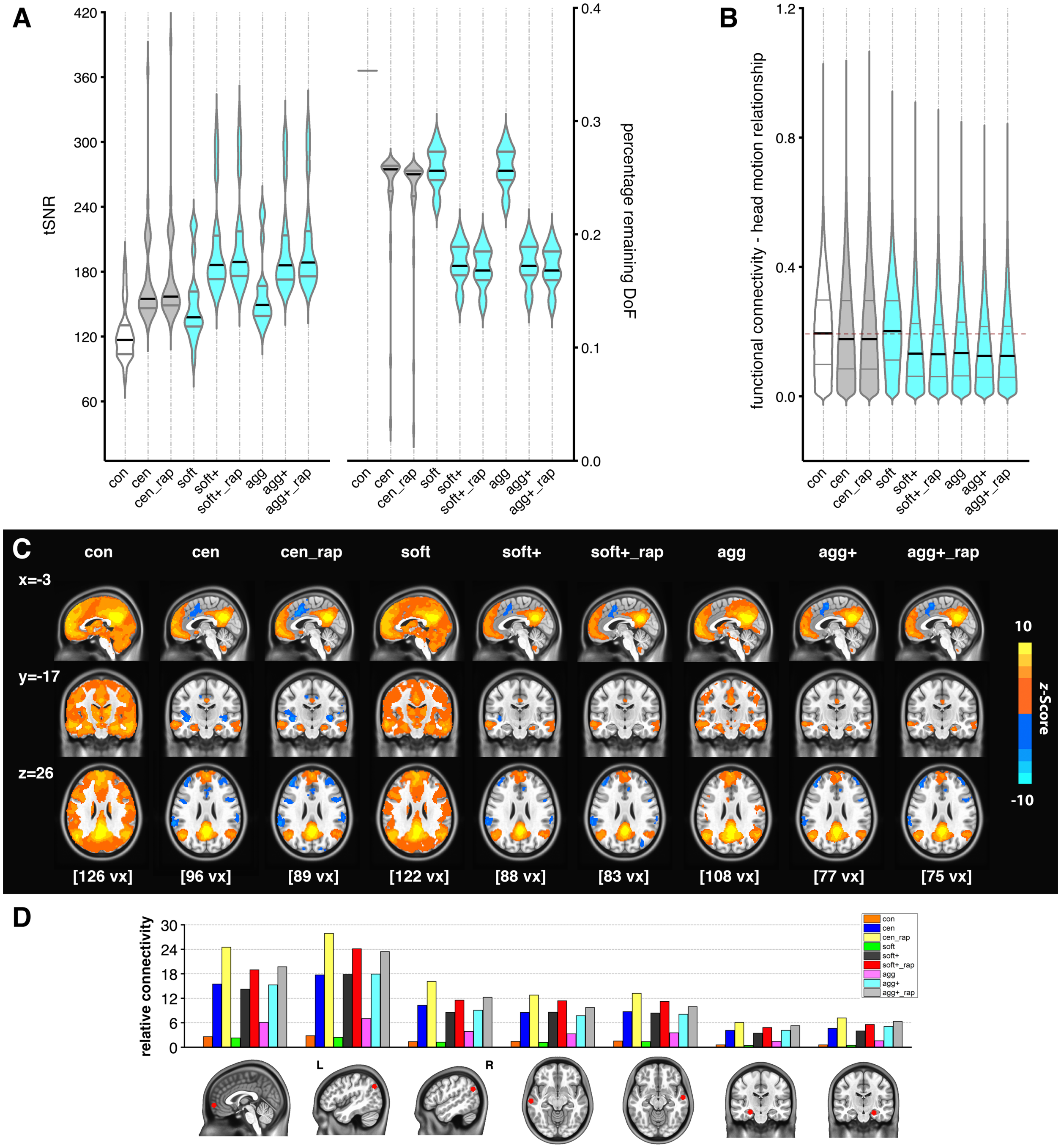

(A) Violin plots of the tSNR and the percentage of remaining DoF across denoising pipelines; **(B)** Violin plots of the relationship between the function connectome and the mean head motion across denoising pipelines; **(C)** PCC functional connectivity pattern obtained from all denoising pipelines, number in the bracket at the bottom of each pipeline denotes the number of voxels needed for the cluster level multiple comparison correction; **(D)** relative connectivity of key ROIs across pipelines. *Abbreviations:* ROI, region-of-interest; PCC, posterior cingulate cortex.

Fig.S3 relationship between working memory activation and task performance (d-prime) of key-ROIs across pipelines

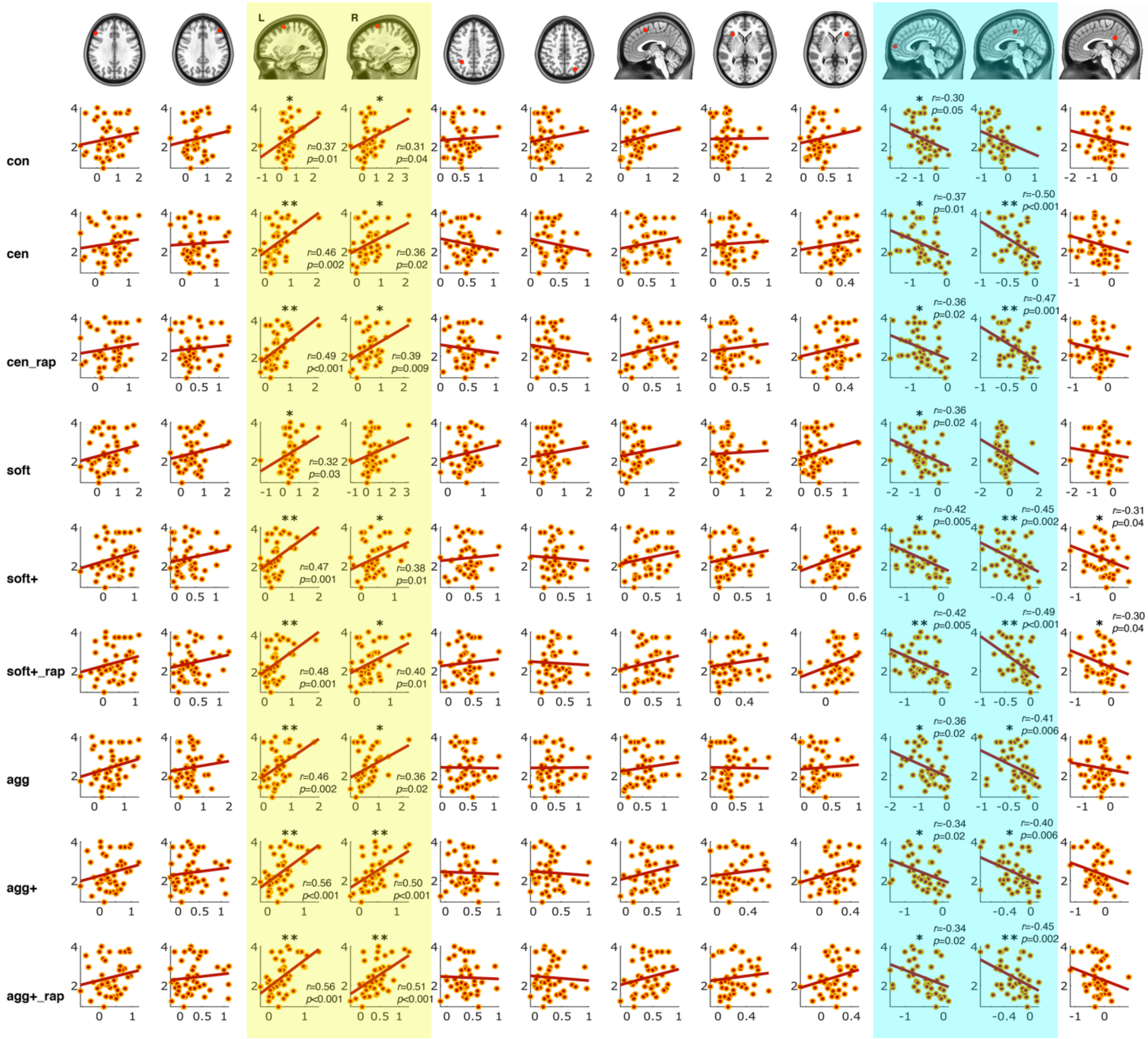

Activated regions with consistent behavioral correlation across pipelines are illustrated in light yellow shaded area and de-activated regions with consistent behavioral correlation across pipelines are illustrated in light cyan shaded area.

Abbreviations: ROI, region-of-interest; *, $p<0.05$; **, $p<0.005$.

Fig.S4 relationship between working memory activation and task performance (reaction time) of key-ROIs across pipelines

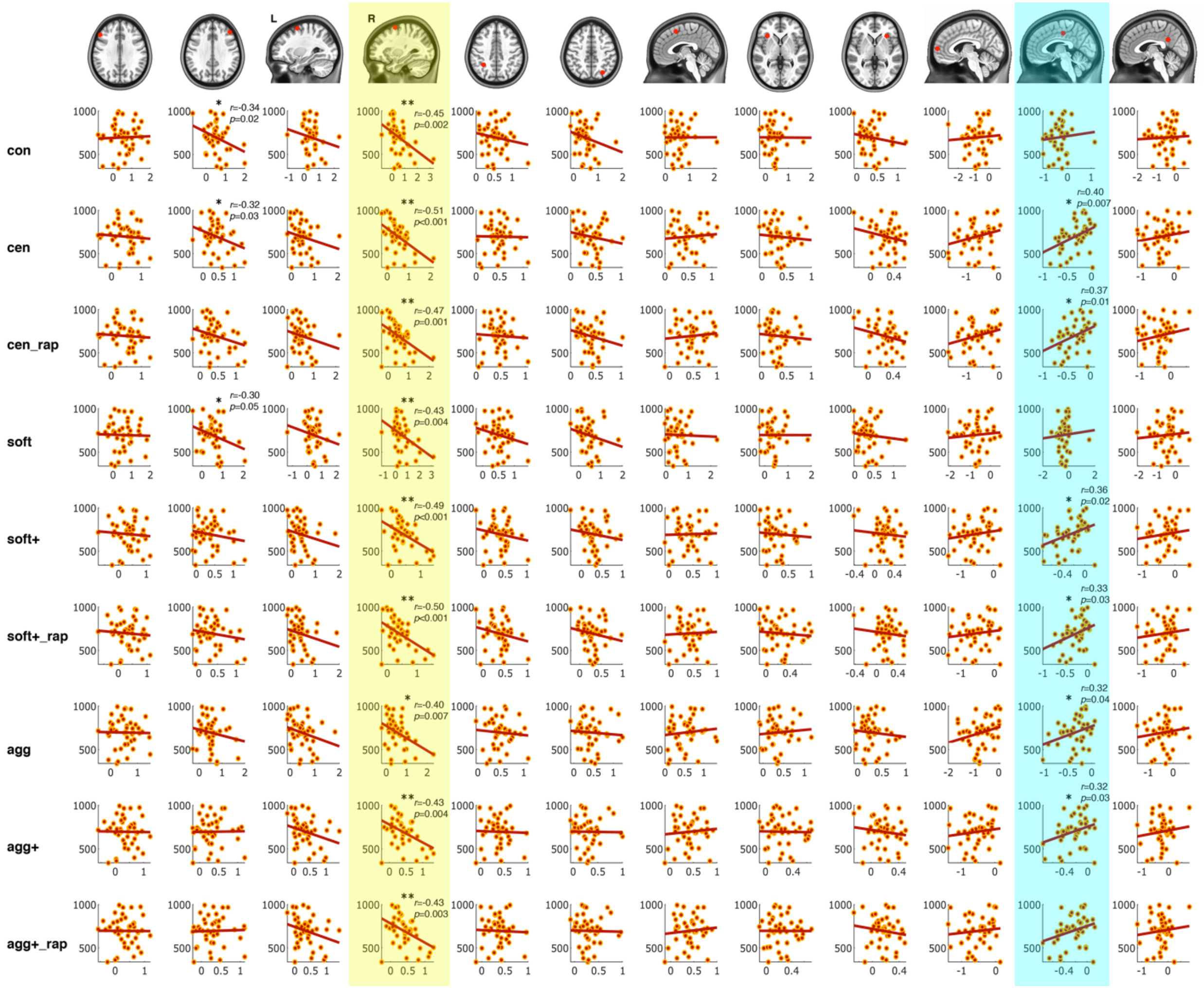

Activated regions with consistent behavioral correlation across pipelines are illustrated in light yellow shaded area and de-activated regions with consistent behavioral correlation across pipelines are illustrated in light cyan shaded area.

Abbreviations: ROI, region-of-interest; *, $p<0.05$; **, $p<0.005$.

Fig.S5 multiband multi-echo resting-state fMRI functional connectivity pattern (PCC) (band-pass version)

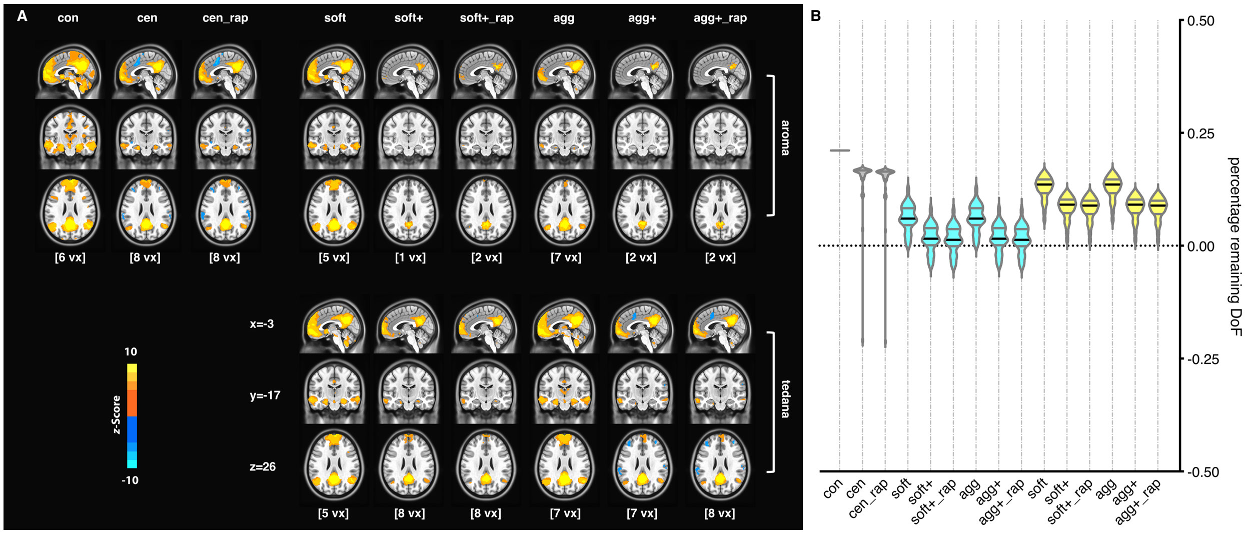

(A) The PCC functional connectivity pattern obtained from all denoising pipelines; the numbers in brackets denote the number of voxels needed for the cluster level multiple comparison correction. **(B)** Percentage of remaining DoF.

Abbreviations: PCC, posterior cingulate cortex; DoF, degrees-of-freedom.

Fig.S6 multiband single-echo resting-state fMRI functional connectivity pattern (PCC) (band-pass version)

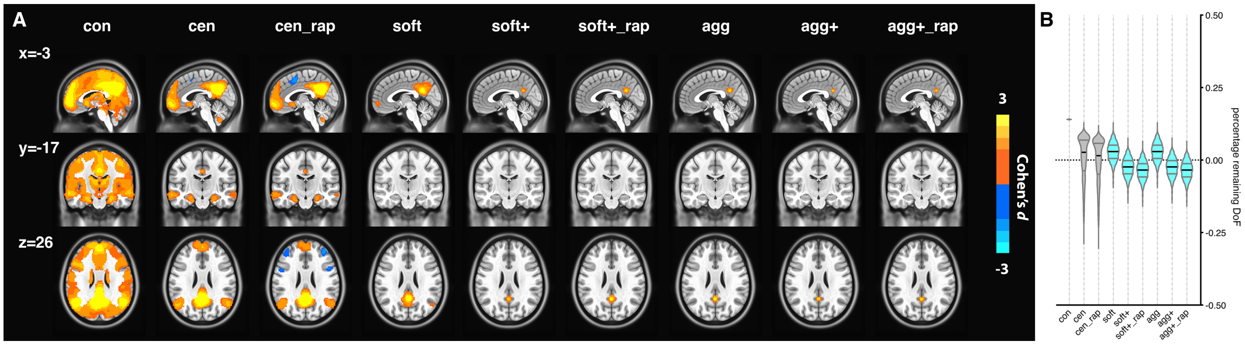

(A) The PCC functional connectivity pattern obtained from all denoising pipelines. **(B)** Percentage of remaining DoF.
Abbreviations: PCC, posterior cingulate cortex; DoF, degrees-of-freedom.

Fig.S7 Effective DoF affects reliable estimation of functional connectivity pattern

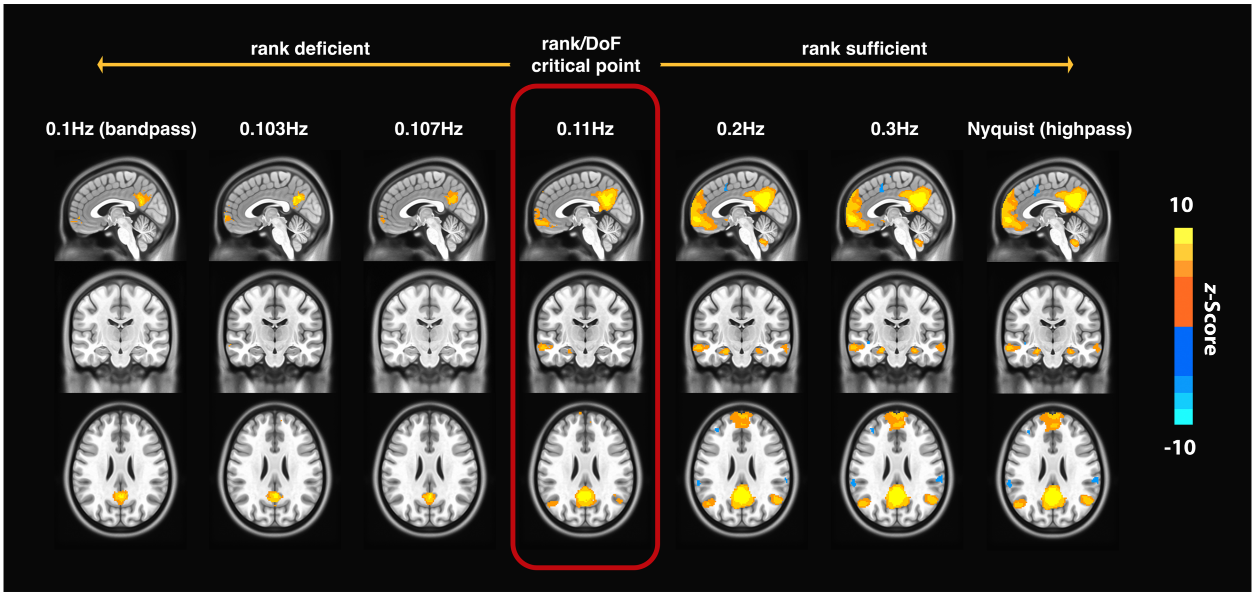

The influence of the effective DoF on the estimation of functional connectivity pattern, using PCC functional connectivity as a demonstration on the multiband multi-echo resting-state fMRI dataset, soft+ pipeline.
Abbreviations: PCC, posterior cingulate cortex.

Fig.S8 control analyses

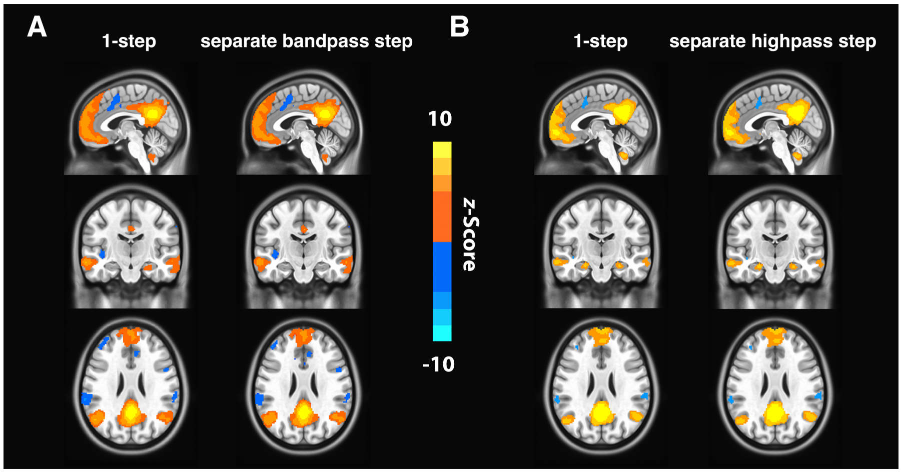

(A) PCC rsFC pattern of 1-step bandpass + nuisance regression vs. sequential bandpass + nuisance regression, when the effective DoF is sufficient (demonstrated with the sbse-rsfMRI data, ‘soft+’ pipeline); **(B)** PCC rsFC pattern of 1-step highpass + nuisance regression vs. sequential highpass + nuisance regression, when the effective DoF is sufficient (demonstrated with the mbme-rsfMRI data, ‘soft+’ pipeline);
Abbreviations: PCC, posterior cingulate cortex; DoF, degrees-of-freedom; GLM, general linear model.

Fig.S9 power spectral density of the denoised time series of the 3 resting-state datasets for all compared pipelines

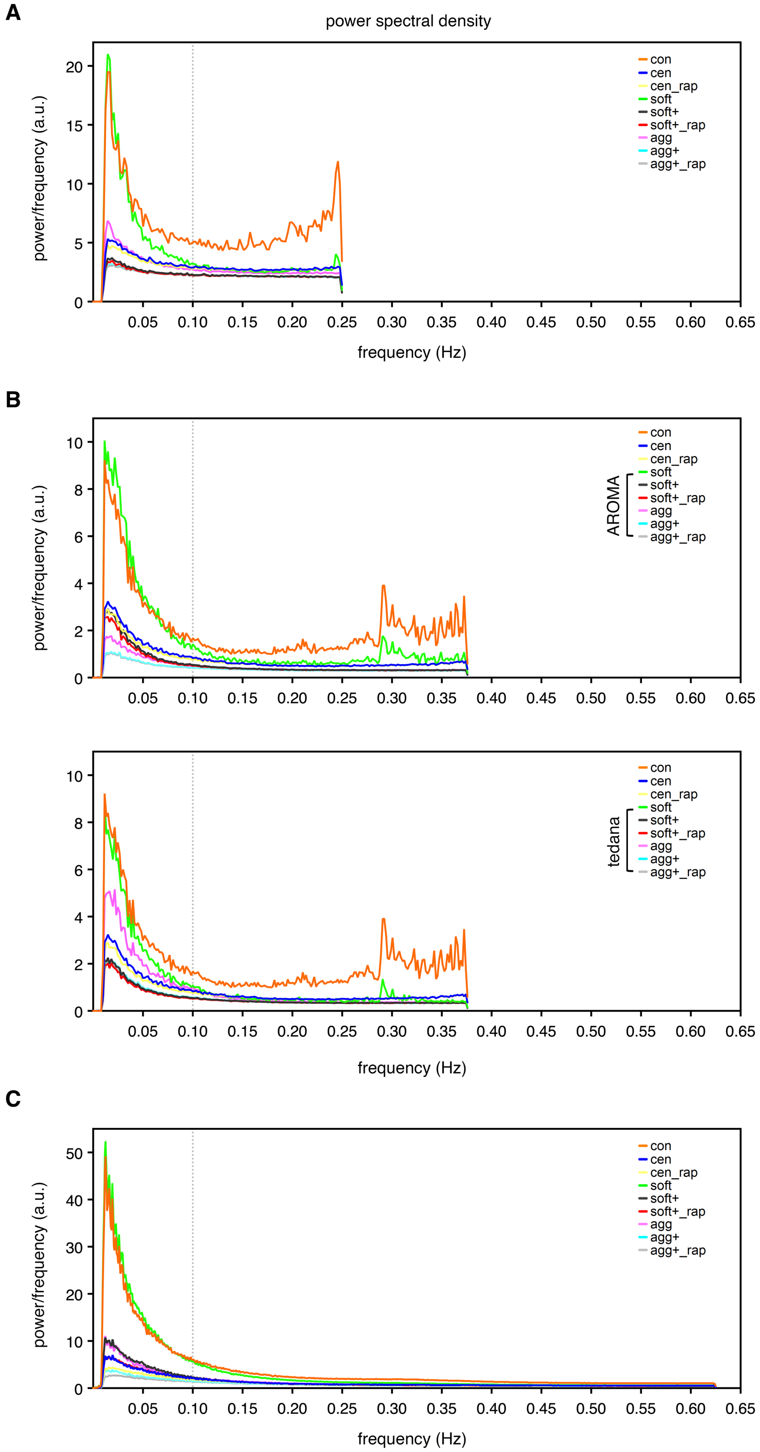

The power spectral density of the single-band single-echo resting-state fMRI data **(A)**, the multiband multi-echo resting-state fMRI data **(B)**, and the multiband single-echo resting-state fMRI data **(C)**. Vertical dashed line indicates the upper cutoff frequency of conventional band-pass at 0.1Hz.

Fig.S10 benchmarking across all denoising pipelines for the multiband single-echo resting-state datasets using an alternative, more stringent Enorm threshold (0.2 mm).

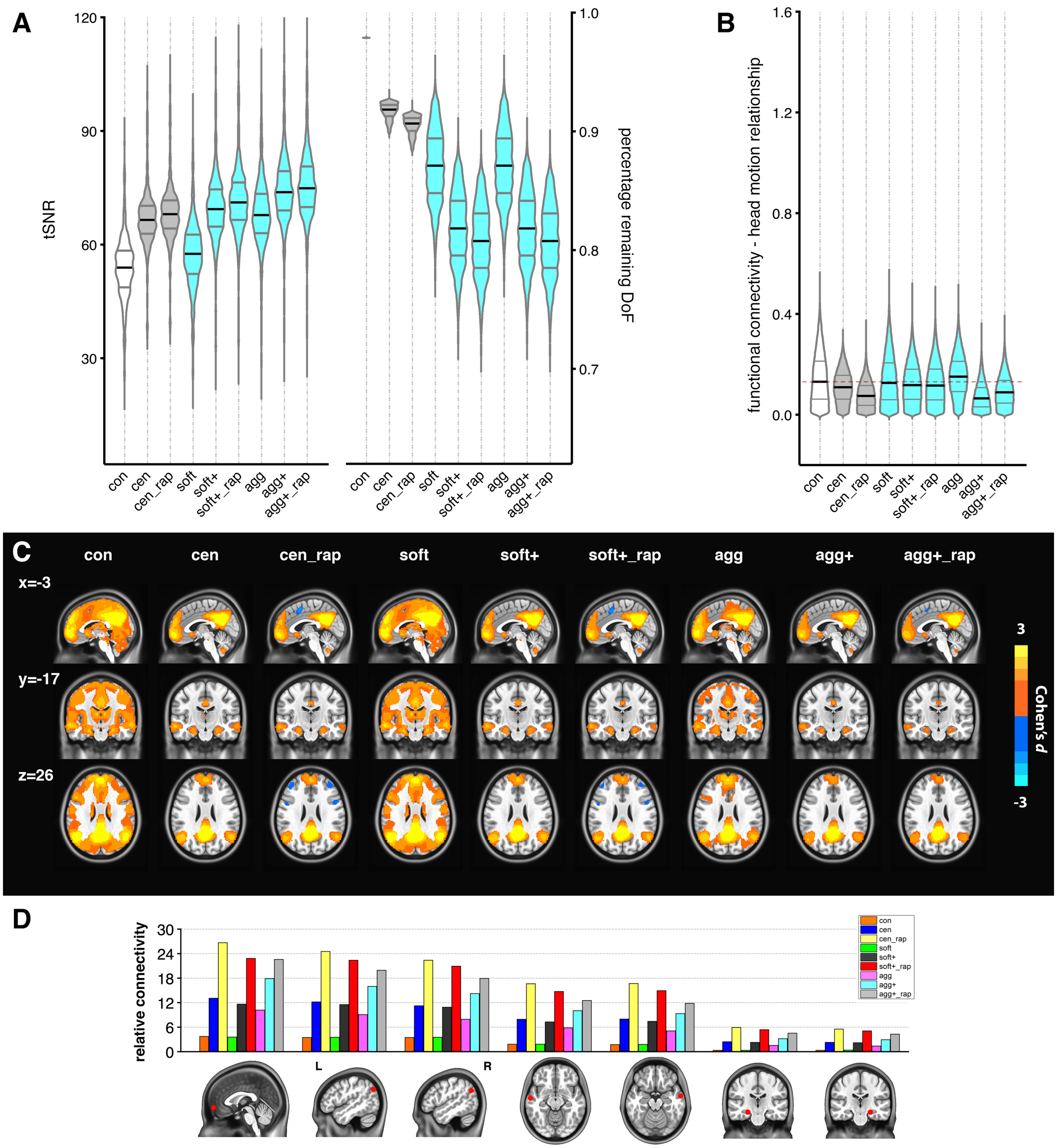

(A) violin plots of the tSNR (higher is better) and the percentage of remaining DoF (higher is better) across denoising pipelines; **(B)** violin plots of the relationship between the function connectome and the mean head motion (lower is better) across denoising pipelines. **(C)** PCC functional connectivity pattern obtained from all denoising pipelines; **(D)** relative connectivity of key ROIs across pipelines (higher is better).

Abbreviations: ROI, region-of-interest; PCC, posterior cingulate cortex.

References:

Forster, S. E., Carter, C. S., Cohen, J. D., & Cho, R. Y. (2011). Parametric Manipulation of the Conflict Signal and Control-state Adaptation. *Journal of Cognitive Neuroscience*, *23*(4), 923-935. https://doi.org/10.1162/jocn.2010.21458
